# Forecasting Root Rot Disease through Predictive Microbial Functional Profiling

**DOI:** 10.1002/advs.202522628

**Published:** 2026-02-17

**Authors:** Chuan You, Peng Ren, Ying Guan, Kai Gong, Zhen Hua, Wuxian Zhou, Xinlan Mei, Yikui Wang, Xiaofang Wang, Yangchun Xu, Qirong Shen, Zhong Wei

**Affiliations:** ^1^ Jiangsu provincial key lab for solid organic waste utilization Key lab of organic‐based fertilizers of China Jiangsu Collaborative Innovation Center for Solid Organic Wastes Educational Ministry Engineering Center of Resource‐saving fertilizers Nanjing Agricultural University Nanjing P. R. China; ^2^ Key Laboratory of Biology and Cultivation of Chinese Herbal Medicines Ministry of, Agriculture and Rural Affairs Institute of Chinese Herbal Medicines Hubei Academy of Agricultural Sciences Enshi Hubei P. R. China; ^3^ Institute of Vegetable Research Guangxi Academy of Agricultural Sciences Nanning P. R. China; ^4^ College of Agro-grassland Science Nanjing Agricultural University Nanning P. R. China

**Keywords:** disease prediction, early diagnosis, machine learning, microbial functional genes, rhizosphere microbiome

## Abstract

Early diagnosis of soil‐borne diseases like root rot is a long‐standing challenge in agriculture. While microbial functional genes are recognized as potent indicators of soil healthy, their application has been primarily limited to current or past soil conditions. Here, we demonstrate that microbial functional genes can transition from descriptive indicators to reliable predictive biomarkers. By analyzing 199 paired metagenomes from healthy and diseased medicinal plants rhizosphere soil samples, we identified a conserved core set of functional genes, specifically those governing biofilm formation, stress response, and plant‐microbe mutualism that are robustly associated with root rot disease. To bridge the gap between discovery and field application, we developed a framework that integrates cost‐effective qPCR assay for these key genes and fused their abundance data with machine learning. This model achieved over 80% accuracy in predicting disease onset from independent, pre‐symptomatic soil samples, identifying risks long before visible symptoms of infection appeared. Our findings suggest a practical path for moving beyond simple microbial correlations toward an active forecasting tool. By positioning microbial functional genes at the core of disease management, this framework provides a targeted approach for mitigating soil‐borne risks and supporting sustainable agricultural practices.

## Graphical abstract

Predicting soil‐borne disease moves beyond observation with a framework that elevates microbial functional genes into reliable forecasting biomarkers. By coupling targeted qPCR assays for core stress‐response genes with machine learning, this method detects root rot risks in pre‐symptomatic soils with over 80% accuracy. The approach offers a scalable, high‐precision tool for preemptive disease management and sustainable crop protection.

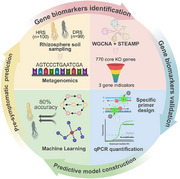

## 1 Introduction

Root rot disease, driven by soil‐borne pathogens like *Fusarium* and *Rhizoctonia*, causes substantial yield losses in high‐value crops, particularly rhizomatous medicinal plants [1, 2, 3, 4]. Due to the concealed nature of early infection below ground, “predictive forecasting” offers far greater practical value for these crops than “retrospective diagnosis” [5]. However, prevailing diagnostic methods primarily target specific pathogens through PCR assays, which often face limitations in field applications [6]. First, pathogen abundance does not always correlate linearly with disease severity; and second, these assays overlook the inherent disease‐suppressive potential of the resident soil microbiome [7, 8, 9]. Second, pathogen populations typically surge only during the disease process, particularly in its mid‐to‐late stages. This presents significant challenges for predicting disease outcomes based on pathogen abundance alone, even in homogeneous soils with identical initial pathogen densities [10, 11]. Therefore, the presence of a pathogen alone often fails to predict disease outcome, creating a pressing need for predictive indicators that reflect the integrated functional state of the soil.

In this context, the rhizosphere microbiome serves as a sophisticated “biosensor” of soil health [12]. A principal limitation of using microbial taxonomic composition for disease prediction lies in its frequent disconnect from functional activities, as knowing “who is there” poorly indicates “what they are doing” in the soil [13]. Furthermore, taxon‐based indicators are highly context‐dependent, as their predictive signals are often confounded and overridden by local environmental conditions, which severely limits the transferability of such diagnostic models across different fields or soil types [14]. Although the taxonomic composition of rhizosphere microbiomes is strongly influenced by soil background and host species, their functional composition remains similar [15]. This suggests the presence of conserved genetic features across rhizosphere ecosystems of various crops. A promising shift moves beyond cataloging microbial taxonomy to profiling their functional traits, as the genes encoding key metabolic processes directly influence plant‐microbe interactions and soil health [16, 17, 18, 19, 20]. For example, the abundance of nitrification genes (e.g., *amoA*) and denitrification genes (e.g., *nirK*, *nosZ*) has been closely linked to nitrogen cycling efficiency and soil health, [21] while the upregulation of biofilm‐related genes (e.g., *pelA*, *epsB*) often correlates with enhanced root colonization and pathogen antagonism [22]. Importantly, shifts in the abundance of these functional genes can signal gut or soil dysbiosis long before visible symptoms of disease emerge [23, 24, 25, 26]. While studies in medical microbiology suggest the potential of functional gene shifts for early disease detection, this concept remains largely unexplored for plant soil‐borne pathogens. Therefore, using the quantitative dynamics of soil functional genes is a promising but underutilized approach for predicting soil‐borne plant diseases.

Despite their potential, a significant translational gap remains between laboratory discovery and real‐world application because current research tends to move along two parallel but disconnected paths. On one hand, discovery‐phase metagenomics powerfully identifies candidate functional biomarkers but is often too costly and complex for routine application, and its findings remain largely correlative, lacking predictive models [16, 27, 28]. On the other hand, targeted assays like qPCR offer the precision and accessibility needs for regular monitoring, but they require researchers to already know exactly which targets to track and what thresholds define a problem [29, 30]. This fundamental disconnect means that functional gene profiles are rarely translated into actionable predictions. In fact, within plant pathology, there is still very limited research that successfully builds robust, field‐deployable disease prediction models using microbial functional gene signatures. Thus, we need a practical and integrated framework that systematically bridges the gap between discovery, validation, and modeling, finally transforming complex biological data into reliable diagnostic tools [31].

Here, we test the hypothesis that a minimal set of conserved microbial functional genes can form the core of a predictive framework for root rot onset. Following the integrated pipeline outlined above, we (i) performed metagenomic sequencing on 199 paired rhizosphere samples to identify robust biomarker candidates; (ii) developed a corresponding qPCR assay for targeted quantification; and (iii) integrated the gene abundance data with machine learning to build a predictive model. Preliminary validation on an independent set of pre‐symptomatic soil samples indicated that this framework could forecast root rot incidence with over 80% accuracy. This work advances the functional gene paradigm from correlation to prediction, establishing a novel, scalable strategy for the early diagnosis of soil‐borne diseases.

## 2 Results

### 2.1 Conserved Genetic Features of the Rhizosphere Microbiome as Stable Indicators of Root Health across Diverse Host and Soils

**FIGURE 1:**
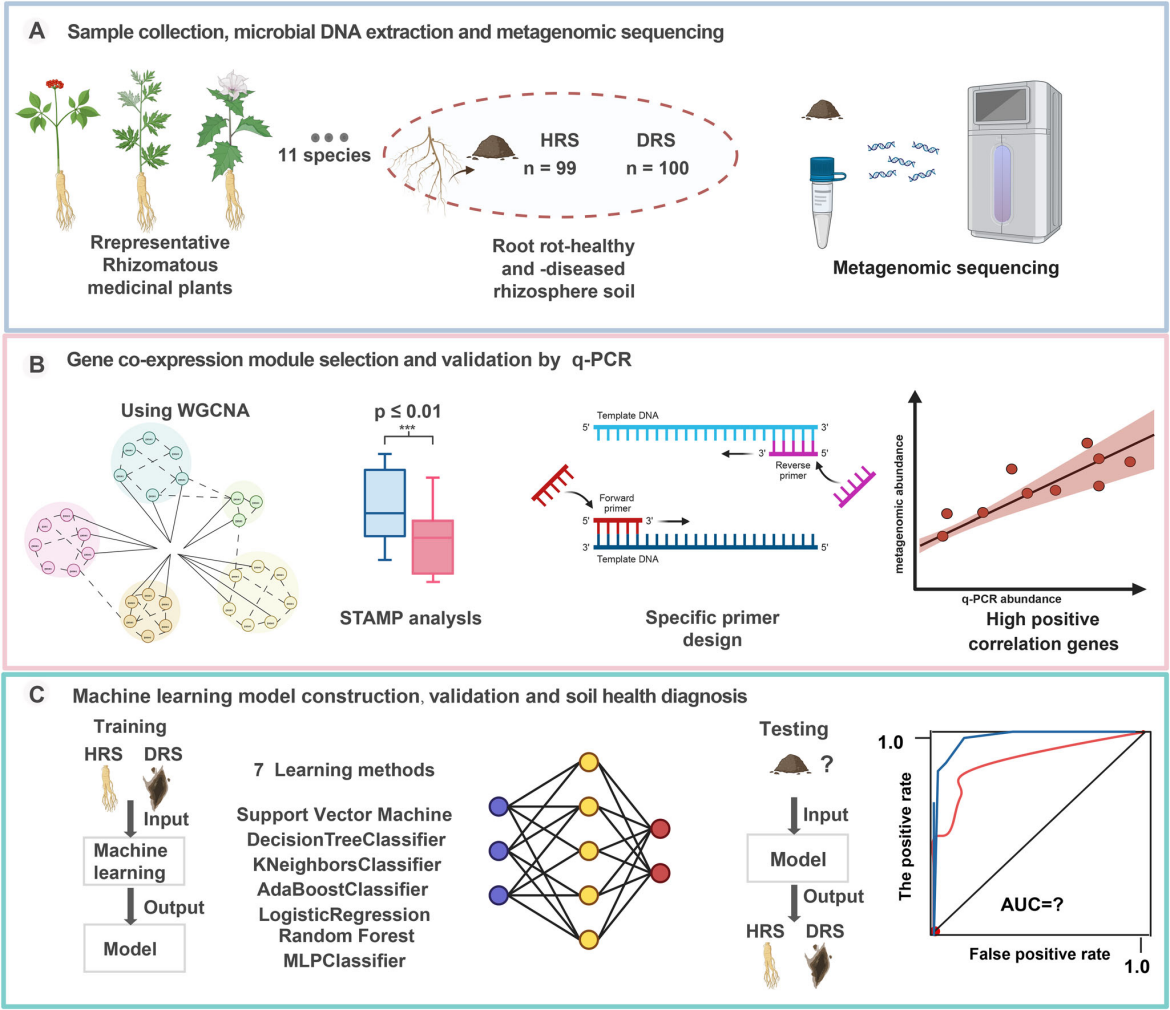
Workflow of the study. (A) Sample collection and metagenomic analysis: Rhizosphere soil samples were collected from paired healthy and diseased plants (n = 199 pairs). Shotgun metagenomic sequencing was performed to profile the microbial functional potential. (B) Identification of key functional genes: Weighted Gene Co‐expression Network Analysis (WGCNA) was used to construct co‐expression modules of KEGG Orthology (KO) genes. Significant Temporal Expression Analysis (STEMP) was further applied to identify key differentially abundant KO genes associated with disease status. Primer design and validation: Specific qPCR primers were designed for the selected KO genes. The specificity and targeting efficiency of each primer set were experimentally validated. (C) Machine learning‐based prediction and validation: Abundance data of key KO genes, obtained via qPCR, were used to train seven machine learning models (support vector machine, decision tree classifier, k‐neighbors classifier, ADA boost classifier, logistic regression, random forest, and MLP classifier). The best‐performing model was independently validated using a separate validation set and an additional pre‐symptomatic soil sample set to evaluate its accuracy in predicting early disease onset.

To identify rhizosphere microbial functional biomarkers for root rot disease, we analyzed a total of 199 paired healthy and diseased rhizosphere soil samples from diverse representative medicinal root and rhizome plants across multiple geographic regions in China. The overall workflow encompassed metagenomic sequencing for biomarker discovery, qPCR validation, and machine learning model development (Figure 1). In total, deep metagenomic sequencing of rhizosphere microbial genomic DNA generated 1545.82 Gbp of clean high‐quality data. An average of 7.77 Gbp clean data were obtained per sample. At the species level, we identified totals of 874 fungal and 48 014 bacterial species in healthy and diseased groups.

**FIGURE 2:**
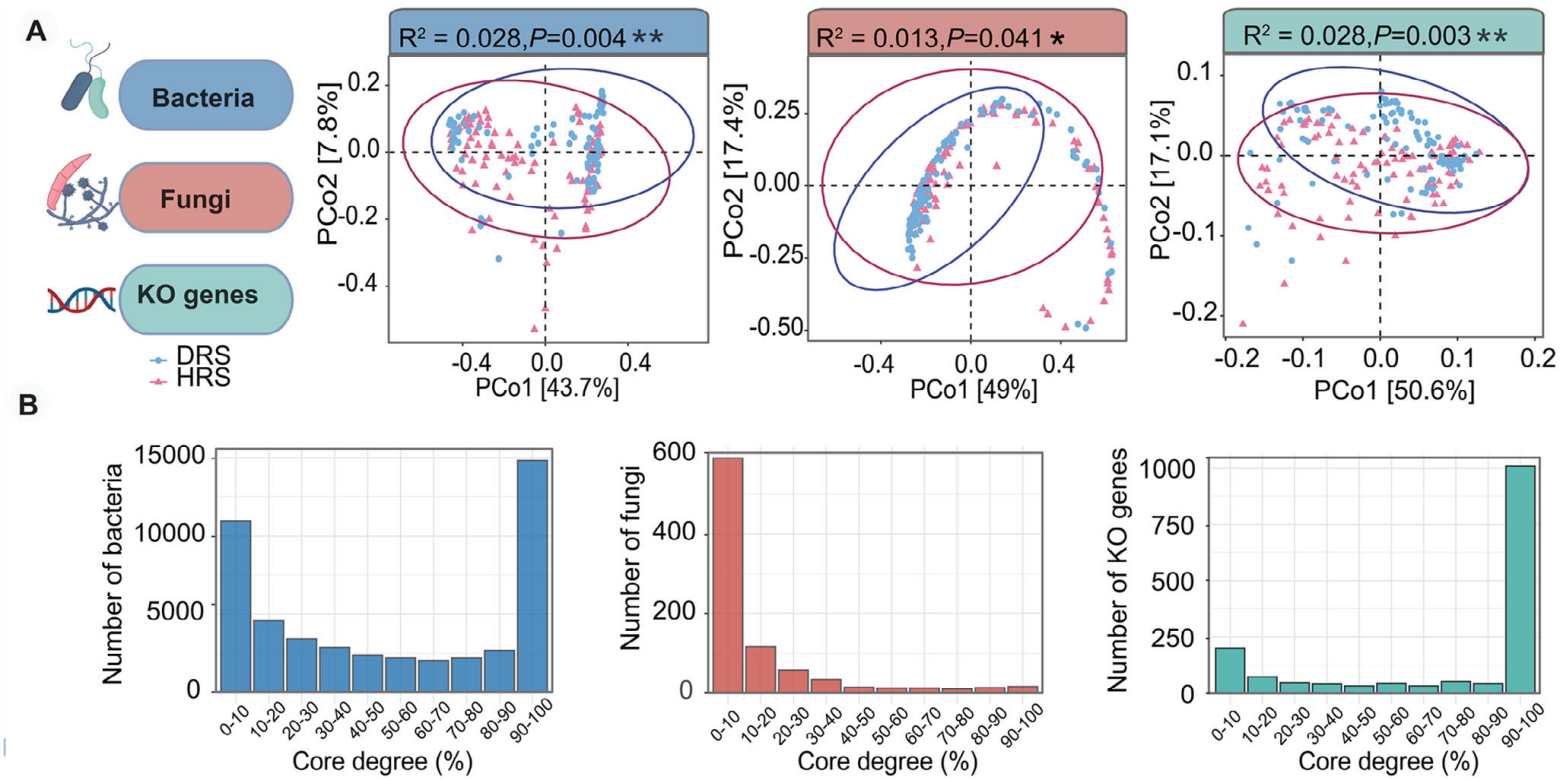
Microbial community and functional gene profiles distinguish healthy (n = 100) and diseased (n = 99) rhizosphere samples. (A) Principal coordinates analysis (PCoA) based on Bray–Curtis distances showing significant separation between healthy and diseased samples across bacterial, fungal, and KO gene profiles. Percentages of variance explained (R^2^) and *p*‐values from ANOVA are indicated for each component. ^*^ *p* < 0.05, ^**^ *p* < 0.01, ^***^ *p* < 0.001. (B) The bar plot shows the core degree of bacterial species, fungal species, and KO genes distributed across ten coreness intervals, defined by their frequency of occurrence across all samples.

The analyses of microbial alpha diversity (Shannon, Species number, Simpson, Evenness and Chao1) at the species level were provided in Tables S2 and S3. All five bacterial indices (Shannon, Species number, Simpson, Evenness and Chao1) and the two fungal indices (Shannon and Simpson) were significantly higher in healthy than these in diseased (Wilcoxon test: *p* < 0.05; Figure S1). Furthermore, a total of 342 pathways and 5897 functional orthologous groups (KOs) were identified from Kyoto Encyclopedia of Genes and Genomes (KEGG). Principal coordinates analysis (PCoA) based on Bray‐Curtis distances revealed that the rhizosphere bacterial, fungal, and KO gene profiles of healthy samples clustered separately from those of diseased samples. PERMANOVA indicated significant differences between healthy and diseased groups for KO genes (R^2^ = 0.028, *p* = 0.003), bacteria (R^2^ = 0.028, *p* = 0.004), and fungi (R^2^ = 0.013, *p* = 0.041, Figure 2A), but not for KEGG pathways (R^2^ = 0.009, *p* = 0.155; Figure S2). Based on PERMANOVA analysis (Figure S3, Table S4), while health status significantly influenced microbial and functional gene profiles, plant species was the strongest determinant of community variation. Geographic location also contributed significantly, with both bacterial and fungal communities showing significant variation across provinces (bacteria: R^2^ = 0.029, *p* = 0.017; fungi: R^2^ = 0.033, *p* = 0.001). In contrast, the functional gene profiles (KO genes) did not exhibit significant geographical differentiation (R^2^ = 0.026, *p* = 0.054) (Figure S3). These results highlight that microbial community structure is more sensitive to geographic variation than functional gene profiles, and underscore the need to account for both host and environmental context when extending this predictive framework. We further analyzed the core degree (defined as the frequency of a gene or species occurring across all samples) of species (bacteria and fungi) and KO genes [32]. Notably, KO genes of the rhizosphere microbiome were overwhelmingly dominated by core genes (core degree: 90%–100%), accounting for 67.5% of all gene entities (Table S5). In contrast, the proportions of core bacterial and fungal species were significantly lower than that of KO genes, at only 27.5% and 3.01%, respectively (Figure 2B; Tables S6 and S7). These findings reveal the conserved nature of microbial functional genes within the rhizosphere microbiome and underscore the importance of establishing health biomarkers derived from functional genes of the rhizosphere microbiota.

### 2.2 Identification of Key Microbial Functional Gene Biomarkers Associated with Root Rot Disease

**FIGURE 3:**
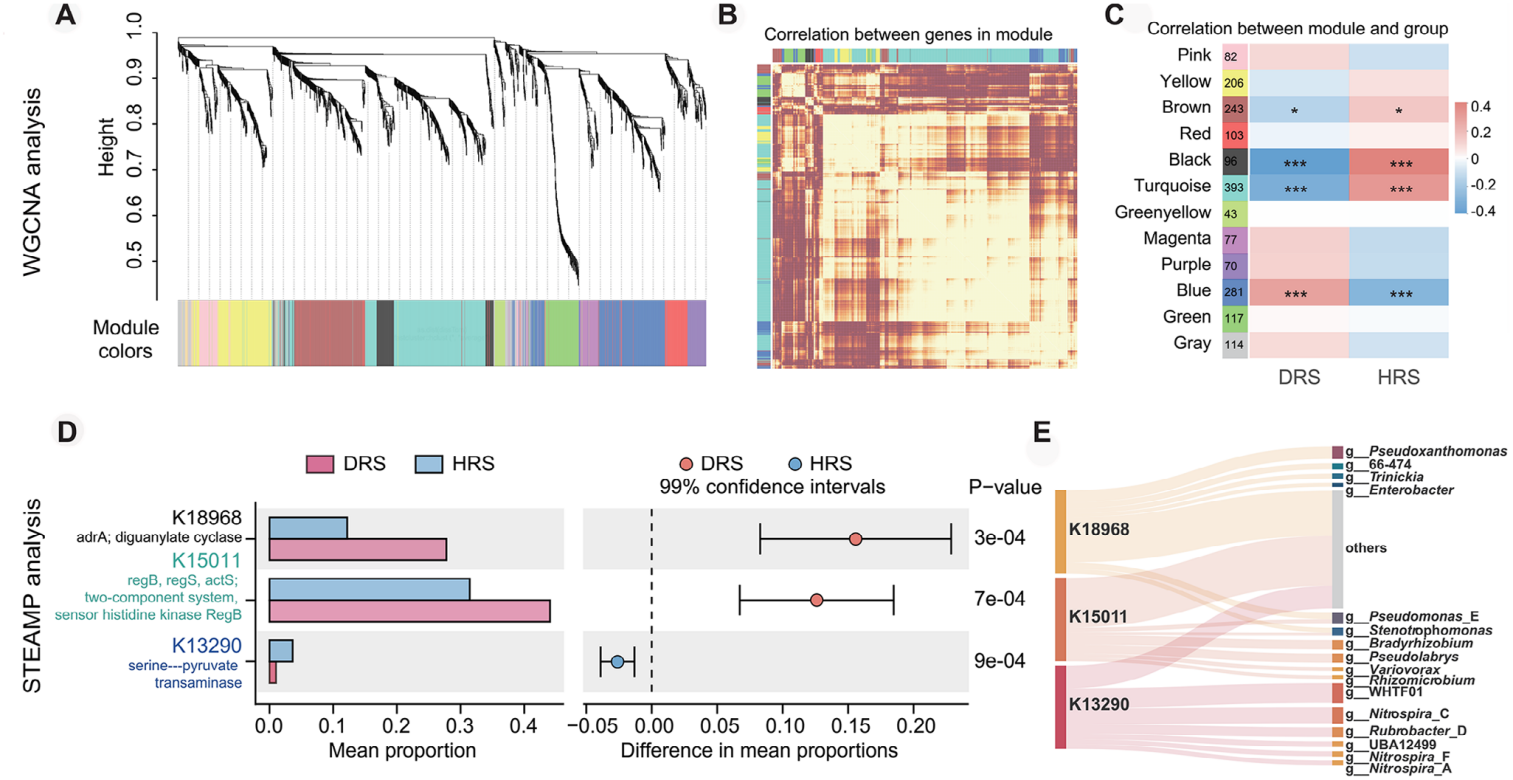
Identification of functional gene modules and key biomarkers associated with rhizosphere health status using Weighted Gene Co‐expression Network Analysis (WGCNA) and Subsequent Significant Temporal Expression Analysis (STEMP) analysis. (A) Clustering dendrogram of KO genes based on Euclidean distance. The upper part represents hierarchical clustering of genes; the lower colored band indicates module assignment, with each color corresponding to a co‐expression module and grey indicating genes not assigned to any module. (B) Heatmap of module eigengene adjacency, depicting co‐expression strength within modules. Darker red indicates higher topological overlap and stronger co‐expression among genes in each module. (C) Module–trait correlations between gene modules and plant health status. Red indicates positive correlation, blue indicates negative correlation. Values represent correlation coefficients with corresponding p‐values in parentheses. ^*^ *p* < 0.05, ^**^ *p* < 0.01, ^***^ *p* < 0.001. (D) STEMP analysis of 770 KO genes from three disease‐associated modules. Left bar plot shows relative abundance of three significantly dysregulated KO genes in healthy (n = 100) vs. Diseased (n = 99) groups; right plot displays 95% confidence intervals of their differential abundance (all *p* < 0.001 after Bonferroni correction). (E) KO genes (K13290, K18968, and K15011) were traced back to genera taxonomy using meta‐linking methods.

We identified functional genes associated with root rot disease using Weighted Gene Co‐expression Network Analysis (WGCNA) on the KO gene abundance profiles derived from the metagenomic data. This analysis resulted in the construction of 12 co‐expression modules (Figure 3A,B). The complete list of KO genes within each module is provided in Table S8. Among these, three modules exhibited exceptionally strong and significant correlations with plant health status (|Correlation Coefficient| > 0.25, *p* < 0.001) and were selected for further investigation (Figure 3C). Subsequent Significant Temporal Expression Analysis (STEMP) was conducted on the gene members of these three disease‐associated modules, which comprised a total of 770 KO genes (Table S9). This analysis initially identified 216 KO genes with nominally significant differential abundance (nominal *p* < 0.05) between healthy and diseased rhizosphere samples. To ensure robustness and control the false discovery rate, we applied a rigorous Bonferroni correction for multiple comparisons with a threshold of *p* < 0.01. This stringent statistical filtering ultimately pinpointed three KO genes (K18968, K15011, K13290) as the most significantly and reliably dysregulated biomarkers. The complete set of 770 genes, including all statistical results from both the initial and corrected analyses, is available in Tables S10 and S11. These three KO genes are involved in critical microbial processes: K18968 (diguanylate cyclase) in biofilm formation, K15011 (two‐component system sensor kinase) in stress and virulence response, and K13290 (serine‐pyruvate transaminase) in amino acid metabolism and plant‐microbe mutualism. Notably, the expression patterns of these three key genes showed a clear dichotomy: genes K18968 and K15011 were significantly enriched in the diseased group (*p* = 3 × 10^−4^ and *p* = 7 × 10^−4^, respectively). In contrast, gene K13290 was markedly enriched in the healthy group (*p* = 9 × 10^−4^, Figure 3D). Furthermore, the taxonomic assignment of these key genes revealed distinct microbial origins. The disease‐associated genes K18968 and K15011 were primarily contributed by bacterial genera known for their versatile metabolism and potential pathogenicity, such as *Pseudoxanthomonas*_A, *Pseudomonas*_E, and *Enterobacter*. In contrast, the health‐associated gene K13290 was predominantly derived from nitrifying and oligotrophic taxa within the *Nitrospira* and *Rubrobacter*_D genera, which are often linked to nutrient cycling and soil ecosystem stability (Figure 3E; Table S12).

Analysis of soil physicochemical properties confirmed that our sample set encompassed a broad range of edaphic conditions (Figure S4), ensuring the representativeness and generalizability of our findings. We measured multiple soil parameters, including pH, cation exchange capacity (CEC), moisture content, available nitrogen (NO_3_^−^─N and NH_4_^+^─N), available phosphorus (AP), available potassium (AK), and DTPA‐extractable calcium (Ca), magnesium (Mg), and sodium (Na). Correlation analysis revealed that the abundance of the three key microbial functional genes was associated with several of these soil properties (Figure S5). Specifically, the contents of DTPA‐extractable Ca, Mg, Na and available K showed negative correlations with the disease‐associated genes (K18968 and K15011) but positive correlations with the health‐associated gene K13290. In contrast, soil moisture, the contents of NH_4_^+^─N, NO_3_^−^─N, and available P exhibited opposite correlation patterns. These observed associations suggest that the local soil environment may influence the functional potential of the rhizosphere microbiome, which could subsequently affect plant health outcomes. The correlation pattern indicates that sufficient availability of certain mineral nutrients (e.g., Ca, Mg, K) is associated with a microbial functional profile characterized by suppressed pathogenic traits and enhanced beneficial functions, while elevated soil moisture and nitrogen/phosphorus levels are linked to an increase in disease‐associated microbial functions. These findings point to potential avenues for further investigation into soil management strategies that could modulate microbial functional traits. Furthermore, we calculated the ratios of the health‐associated gene to each disease‐associated gene (K13290/K15011 and K13290/K18968). These ratios effectively capture the relative change and imbalance between opposing functional potentials (beneficial vs pathogenic) in the microbiome, providing a complementary perspective to the individual gene analyses. Notably, the correlations of these ratios with soil physicochemical properties (Figure S5) exhibits consistent trends with those of K13290 alone, underscoring the stability of this functional relationship.

### 2.3 Validation of Key Functional Gene Biomarkers Using Q‐PCR

**FIGURE 4:**
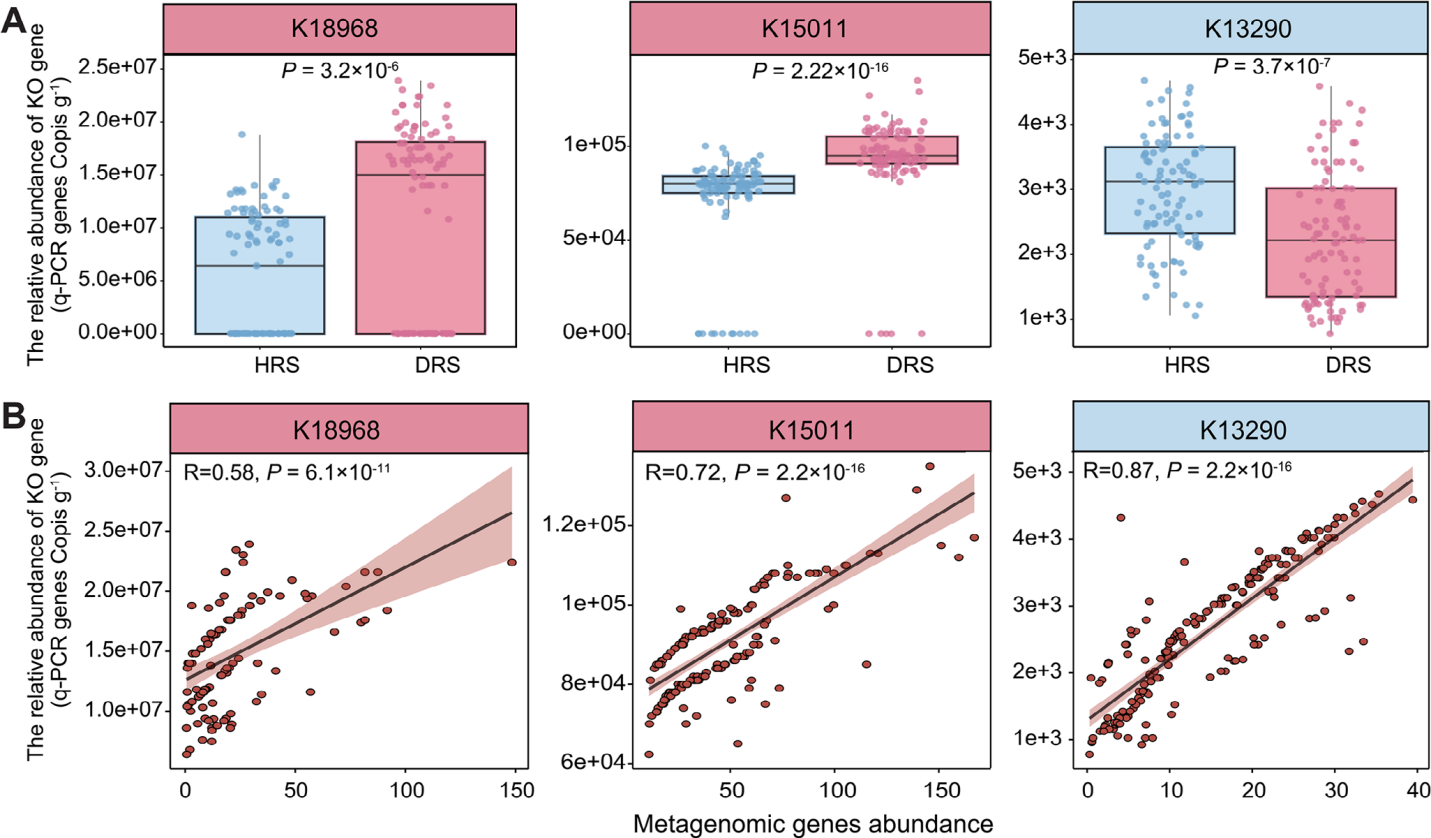
Validation of key microbial gene biomarkers by qPCR and correlation with metagenomic profiles. (A) Box plots showing the relative abundance of three KO genes (K18968, K15011, K13290) measured by qPCR in healthy (HRS, n = 100) and diseased (DRS, n = 99) rhizosphere soil samples. Statistical significance was determined by the Wilcoxon rank‐sum test. (B) Spearman correlation analysis between gene abundance values obtained from metagenomic sequencing and qPCR (n = 199).

To translate metagenomically identified genetic biomarkers into a deployable diagnostic tool, we developed a quantitative PCR (qPCR) assay targeting three KO genes (K18968, K15011, K13290) strongly associated with root rot disease status. We quantified the abundance of these genes in all 199 paired healthy and diseased rhizosphere soil samples. The qPCR results robustly confirmed the trends observed in the metagenomic sequencing data: the two disease‐associated genes (K18968 and K15011) showed significantly higher abundance in the diseased group (*p* < 0.01), whereas the health‐associated gene (K13290) was significantly more abundant in healthy samples (Figure 4A). Furthermore, the quantitative results from qPCR demonstrated a strong and statistically significant positive correlation with the abundance values derived from metagenomic sequencing (Spearman correlation, R^2^ = 0.72, *p* < 0.001; Figure 4B). This high level of agreement validates the qPCR assay as a reliable, accurate, and cost‐effective method for quantifying these key microbial functional gene markers in complex soil environments. In contrast, quantification analysis of rhizosphere *Fusarium oxysporum* abundance via qPCR (n = 199) revealed no significant difference between healthy and diseased rhizosphere samples (*p* = 0.511; Figure S6). These results confirm that qPCR is a reliable and efficient method for quantifying microbial functional genes in complex soil matrices. Moreover, the abundance of these functional genes served as a more effective indicator for distinguishing between healthy and diseased plants compared to the density of the pathogen itself.

### 2.4 Diagnosis and Early Prediction of Root Rot Disease Using Microbial Functional Gene‐Based Machine Learning Models

**FIGURE 5:**
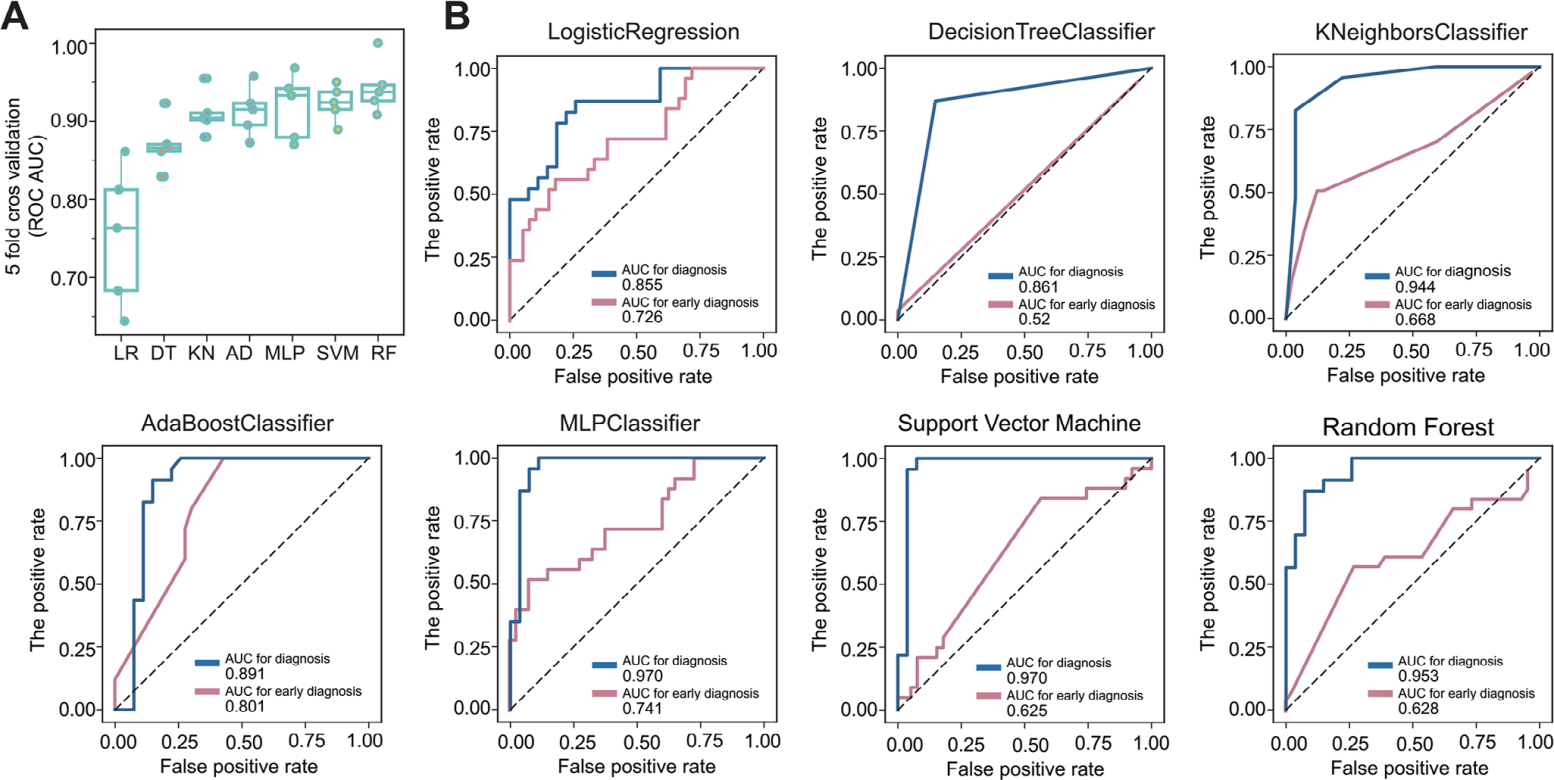
Performance evaluation of machine learning models for predicting root rot disease. (A) Model comparison on the hold‐out test set. Receiver Operating Characteristic (ROC) curves for the seven machine learning models trained on qPCR data (n = 199 samples, 7:3 split). (B) Comparative ROC analysis on independent validation sets. ROC curves showing the performance of the top‐performing models when applied to two independent validation sets: the hold‐out test set (n = 60, solid lines) for disease diagnosis validation and the pre‐symptomatic soil set (n = 64, dashed lines) for early prediction assessment.

To develop a predictive model for root rot disease, we utilized the qPCR abundance data for the three key biomarker genes (K18968, K15011, K13290). The dataset of 199 samples was randomly divided into a training set (70%) and an independent testing set (30%). We evaluated the performance of seven machine learning models using 5‐fold cross‐validation on the training set. Among them, the Random Forest and Support Vector Machine (SVM) models exhibited superior performance in distinguishing healthy versus diseased samples in the testing set, achieving high average AUC values of 0.953 and 0.970, respectively (Figure 5A; Table S13). Their robust performance and interpretability (especially for Random Forest) led us to highlight these two models as primary candidates for diagnostic applications where high accuracy is paramount. In addition, to benchmark the predictive power of microbial functional genes against traditional soil metrics, we constructed comparative models using only the nine measured soil physicochemical properties set. For comparison, models using only nine soil physicochemical properties were built. They achieved lower test‐set AUC values (0.61–0.81) than models based solely on microbial functional genes (Figure S7, Tables S13 and S14), demonstrating the superior predictive power of the functional gene set.

To further assess the model's capability for early prediction, we established an independent early‐stage external dataset comprising 64 regional geographic diversity soil samples. The soil samples were collected from multiple plots across several counties (including Dingxi, Longxi, and Minxian), which from the main traditional cultivation area for *Angelica sinensis* and *Astragalus membranaceus* in Gansu Province (Table S1). This sampling strategy captured the regional soil diversity within this key agro‐ecological zone. A total of 64 samples were taken from fields before planting. Their functional gene profiles were analyzed to generate predictions, which were later validated against the health outcomes of plants grown in the corresponding soils. The health status (healthy or root rot symptomatic root) of the subsequently planted *Angelica sinensis* and *Astragalus membranaceus* plants was meticulously recorded. At the end of the greenhouse experiment, plants were categorized as either diseased (n = 39) or healthy (n = 25) based on the health status. Interestingly, when the qPCR data from this external data set was applied to the trained models, MLP, SVM, and RF failed to demonstrate strong early diagnostic capability. In contrast, the AdaBoost Classifier emerged as the only model to achieved the best performance, with an AUC of 0.801 (Figure 5B; Table S13). This result underscores its robustness and generalizability for pre‐symptomatic prediction across diverse soil samples.

## 3 Discussion

In this study, abundance of the pathogen *Fusarium oxysporum* itself showed no significant difference between healthy and diseased rhizosphere samples, indicating that pathogen abundance alone is insufficient for predicting plant health status. This inconsistency may stem from the complex soil matrix, where microbial functionality and interactions rather than mere pathogen presence dictate disease outcomes [33]. Soils often harbor diverse microbial communities that can suppress pathogen virulence through competition or antagonism, thereby decoupling pathogen density from disease severity [34]. Such functional redundancy and context‐dependent pathogenicity highlight the limitation of pathogen‐centric diagnostics and underscore the necessity of functional gene‐based indicators that integrate microbial ecological dynamics. This shift is supported by the ecological principle that disease outcomes may be influenced not merely by the pathogen, but by the functional structure and stability of the entire microbiome. The growing recognition that microbial functions, rather than taxonomic composition, are increasingly recognized as potentially more robust biomarkers for soil health assessment [35]. The strong separation between healthy and diseased samples in PCoA based on KO genes (R^2^ = 0.028, *p* = 0.003) further supports the predictive potential of functional genes in diagnosing soil‐borne diseases. This aligns with the growing consensus that functional profiles often better reflect soil microbial status than taxonomic data alone. Thus, our model provides a more robust and generalizable diagnostic tool that transcends pathogen‐centric limitations. This contrast highlights a key advantage of our functional approach: it captures the integrated pathogenic potential and disease‐suppressive capacity of the entire microbiome, offering a more holistic and predictive measure of soil health than pathogen‐centric diagnostics. Our functional gene‐based forecasting framework occupies a distinct niche among existing diagnostic tools (a comparative summary is provided in Table S16). Unlike pathogen‐specific qPCR, it assesses the functional potential of the entire microbiome, integrating disease‐suppressive capacity. Compared to discovery‐phase metagenomics, it translates insights into a cost‐effective, routine‐ready qPCR assay. Relative to machine learning models based on taxonomic amplicon sequencing, models built on conserved functional genes may offer greater transferability across diverse soils, as functional profiles are often more stable than taxonomic compositions. While soil physicochemical models are easily obtained, our approach incorporates direct biological mechanisms, yielding superior predictive accuracy.

The three identified KO genes: K18968 (*adrA*; diguanylate cyclase), K15011 (*regB*, *regS*, *actS*; two‐component system sensor histidine kinase RegB), and K13290 (serine‐pyruvate transaminase) represent critical functional pathways involved in microbial adaptation and plant‐microbe interactions. K18968, involved in biofilm formation through diguanylate cyclase activity, was significantly enriched in diseased samples. This gene facilitates bacterial attachment and biofilm formation, potentially enhancing pathogen persistence in the rhizosphere [36, 37]. Similarly, K15011, encoding a two‐component system sensor kinase, regulates microbial stress responses and virulence factor expression, contributing to pathogenic adaptation in diseased soils [38, 39]. In contrast, K13290, involved in glycine, serine and threonine metabolism, was markedly enriched in healthy samples. This gene may support plant health through multiple mechanisms, including synthesis of protective metabolites, modulation of plant defense responses, and maintenance of microbial community stability [40]. The enrichment of K13290 in healthy plants strongly suggests its role in sustaining beneficial plant‐microbe mutualisms. Its quantification thus provides a direct readout of a soil's capacity to support plant health, a core tenet of soil health assessment. Furthermore, the negative correlations between Ca, Mg, Na, K and disease‐associated genes (K18968 and K15011), alongside their positive correlation with health‐associated K13290, suggest a suppressive effect of these minerals on pathogenic traits and a stimulatory effect on beneficial functions. Specifically, K18968 and K15011 are linked to pathogen persistence and adaptation. It is plausible that adequate Ca and Mg availability strengthens plant cell walls and enhances defense signaling, thereby reducing the ecological advantage of biofilm‐forming or virulence‐activated pathogens [41]. Conversely, K13290 contributes to glycine and serine metabolism, which supports the synthesis of protective metabolites and promotes microbial mutualisms. Higher K^+^ and Ca^2^^+^ availability may facilitate these beneficial metabolic pathways, reinforcing plant–microbe cooperation and system stability. In contrast, soil water, NH_4_^+^─N, NO_3_^−^─N, and P exhibited opposite correlation patterns, being positively associated with disease‐linked genes and negatively with K13290. Excessive soil moisture likely creates hypoxic conditions that favor biofilm‐forming pathogens utilizing K18968, while high nitrogen and phosphorus levels may disrupt microbial stoichiometry, upregulating stress‐sensing pathways like K15011 and suppressing central metabolic enzymes such as K13290 [42, 43]. These correlative findings point to the possibility that soil amendment strategies, such as optimizing water drainage, balancing fertilizer use, and supplementing key minerals, could potentially modulate microbial functional gene expression, influence pathogenicity traits, and promote beneficial microbial metabolism, suggesting a promising avenue for future research into managing soil health through microbial functional ecology.

Our study bridges the critical gap between the potential of microbial functional genes as indicators, as discussed by Jia et al., [16] and their practical application as predictive tools for soil health management. Our qPCR assay successfully validated the metagenomic findings, showing strong correlation between sequencing and quantitative PCR data (R^2^ = 0.72, *p* < 0.001; Figure 3B). This suggests that targeted quantification of key functional genes could provide a reliable, cost‐effective alternative to complex metagenomic analyses for routine monitoring. The consistency between these methods supports the potential robustness of our identified biomarkers and suggests their possible suitability for field application. This approach aligns with the growing emphasis on developing practical monitoring tools that can bridge the gap between scientific understanding and agricultural application. The integration of cost‐effective qPCR quantification with machine learning is the cornerstone of our predictive framework. This synergy transforms static gene abundance data into a dynamic forecasting tool, moving beyond description to prediction. The superior performance of Random Forest and SVM models (AUC: 0.953‐0.970) in distinguishing healthy and diseased samples highlights the power of ensemble learning and kernel‐based methods in handling microbial abundance data for diagnostic purposes [44, 45]. Notably, the AdaBoost classifier emerged as the most effective model for early prediction (AUC = 0.801) on the independent validation across diverse soil samples (Figure 5B). AdaBoost demonstrated superior performance in early disease prediction, likely due to its ability to detect subtle microbial gene patterns within complex soil data and its relative robustness to class imbalance. These characteristics make it particularly suitable for identifying pre‐symptomatic soil conditions where biological signals are weak and often confounded by environmental variation [46]. Our analysis reveals a clear scenario‐dependent applicability: while MLP, SVM, and Random Forest excelled in internal testing, AdaBoost exhibited the strongest generalization to the external validation set. Therefore, AdaBoost is recommended for early‐risk forecasting where robustness to new data is critical, whereas models like SVM and Random Forest may be reserved for contexts where internal performance is the sole priority. The success of machine learning approaches in our study supports their increasing application in microbial ecology for predicting complex biological outcomes.

We developed a practical pipeline that translates microbial functional gene signatures into a soil health diagnostic tool. This workflow effectively translates complex metagenomic insights into a simple, qPCR‐based assay empowered by AI, making predictive soil health assessment accessible and practical. The ability to accurately predict disease onset using early soil samples (before plant symptom development) offers unprecedented opportunities for preventive management. However, several considerations warrant attention for practical implementation. The geographical variability of soil microbiomes may require region‐specific model calibration, [47] while seasonal fluctuations in microbial communities necessitate temporal validation [48]. Future studies should explore the integration of environmental parameters with microbial data to enhance model robustness across diverse agricultural systems. Research has shown that soil microbiomes can exhibit functional redundancy across different environments, but local adaptation factors must be considered for effective application. Collectively, our work demonstrates that microbial functional genes are not merely passive indicators reflecting soil status; they are active, quantifiable components that can be harnessed to build powerful predictive models. We have thus advanced the concept put forward by Jia et al., that functional genes are effective indicators by proving they can form the basis of effective predictors [16]. The significance of our study is twofold: it provides strong validation for microbial functional genes as the most effective predictors of soil health to date, and it delivers a replicable, quantifiable, and scalable framework for the early diagnosis of soil‐borne disease. A practical advantage of this framework is its foundation on qPCR rather than discovery‐phase metagenomics. This makes the diagnostic assay genuinely cost‐effective for potential routine use, with per‐sample costs substantially lower than sequencing and results obtainable within hours. The protocol is compatible with standard molecular lab infrastructure, suggesting a realistic pathway toward a commercial diagnostic service or kit, although its operational success will depend on further validation of model transferability across regions to ensure the cost‐benefit advantage is maintained. While developed for root rot in medicinal plants, the core principle of our framework by predicting disease risk via conserved microbial functional traits, holds promise for broader application. Many soil‐borne diseases (e.g., *Fusarium* wilt, *Phytophthora* root rot) involve similar microbial warfare for root colonization. The general processes our genes represent (biofilm‐based persistence, environmental stress sensing, and host‐compatible metabolism) are universal. Adapting this framework to other crops or pathogens would require identifying a minimal, conserved functional gene set specific to that pathosystem through a similar discovery pipeline, followed by analogous qPCR assay development and model training. This underscores the generalizability of the approach, while acknowledging the need for target recalibration for each major disease complex.

By integrating metagenomic screening, qPCR‐based quantification, and machine learning modeling, this study has developed a holistic approach that can predict disease onset with over 80% accuracy, showing potential as a practical tool for sustainable agricultural management. In summary, while related studies powerfully demonstrate the utility of microbial community data and machine learning, our work advances the paradigm by (i) focusing on mechanistically informative functional genes rather than taxonomic or general metagenomic profiles, (ii) establishing a complete translational pipeline from discovery to a potential field‐deployable qPCR assay, and (iii) providing robust validation of pre‐symptomatic prediction using a minimal set of conserved biomarkers. These points, along with a direct citation of the relevant comparative study, are now presented in the manuscript to better position our work within the field. We anticipate that this model, which directly leverages the predictive power of microbial functional genes, will become a foundational component in the next generation of soil health evaluation and precision agriculture.

## 4 Experimental Section

### 4.1 Plants and Samples Collection

In October 2023, 11 rhizomatous medicinal plants (Angelica sinensis, Astragalus membranaceus, Codonopsis pilosula, Pinellia ternata, Panax notoginseng, Platycodon grandiflorus, Polygonatum sibiricum, Salvia miltiorrhiza, Angelica dahurica, Atractylodes macrocephala and Saposhnikovia divaricata) were obtained from four provinces (Gansu, Anhui, Yunnan, and Hebei) in China where the representative production area is located (Figure 1A; Table S1). Five representative subplots were sampled from each field. Within each subplot, plants with both healthy and root rot symptoms of root rot disease were sampled and pooled as a composite sample. A total of 100 healthy and 99 (one diseased sample was excluded due to insufficient soil DNA yield) diseased plant samples were collected from 20 different sampling points across 20 large‐scale fields. Sampling Information for all samples is provided in Table S1. Rhizosphere soil collection was performed as described previously [49]. In brief, the samples were prepared by gently shaking the roots, ensuring that approximately 1 mm of rhizosphere soil remained intact. The roots were then subjected to vortexing in PBS buffer (0.1 m phosphate buffer, 0.15% Tween 80, pH 7.0) for 4 min, with the process repeated twice. The resulting suspension was filtered through an 80‐mesh sieve to separate root debris, followed by centrifugation at 4000 × g for 10 min. The small particulate matter obtained after centrifugation was retained as the rhizosphere soil fraction.

### 4.2 Metagenomic Sequencing

Rhizosphere samples from both healthy and diseased soil were collected and subjected to metagenomic shotgun sequencing. In total, 199 rhizosphere soil samples were obtained and subjected to metagenomic sequencing analysis, to identify and validate rhizosphere microbial functional biomarkers for root rot disease diagnosis. Each of these 199 samples represents an independent biological replicate. For each sampled plant, soil from multiple root segments was collected and homogenized to form a single, composite rhizosphere sample, ensuring a representative profile for that individual. No pooling of soil across different plants was performed at any stage. Both healthy and diseased rhizosphere soil samples were collected for DNA extraction, amplicon sequencing, and analysis. Genomic DNA was extracted from each soil sample using the DNeasy PowerSoil Kit (QIAGEN GmbH, Germany), following the manufacturer's protocol. DNA concentrations were measured using a Qubit 2.0 fluorometer (Thermo Fisher, USA), and the samples were diluted to achieve consistent concentrations across all rhizosphere DNA samples. Shotgun sequencing was conducted by Biozeron (Shanghai, China) using the Illumina HiSeq platform. The resulting metagenome reference gene set was aligned with the NCBI NR database (June 2020) using BLASTP for taxonomic assignment. Gene annotations were retrieved from the Kyoto Encyclopedia of Genes and Genomes (KEGG) database (Release 95.2) to identify associated pathways. Additionally, the gene sequences were functionally profiled using the NCycDB database, a manually curated repository specializing in nitrogen cycling genes [50]. Gene abundance was then calculated and normalized using the Reads Per Kilobase per Million mapped reads (RPKM) method to account for variations in gene length and sequencing depth across samples. To correlate species information with functional data, the meta‐links method was used, linking open reading frames (ORFs) with matching identifiers (ORF IDs) [51]. To account for the inherent variability of field‐collected rhizosphere microbiomes, the study was designed and analyzed as follows. The sampling strategy included a wide range of host plant species and geographical locations. For bioinformatic analysis, Weighted Gene Co‐expression Network Analysis (WGCNA) was applied to identify co‑expression gene modules. Subsequently, Significant Temporal Expression Analysis (STEMP) was performed, and a strict Bonferroni correction (*p* < 0.01) was applied to identify differentially abundant genes.

### 4.3 Measurement of Rhizosphere Soil Physicochemical Properties

The physical and chemical properties of the soil were measured in healthy and diseased soil samples harvested in the field, as mentioned above. For pH analysis, soils were extracted in deionized water for 1 h to achieve a soil: solution ratio of 1: 2.5. The water pH was subsequently measured using a combination pH electrode. For moisture content analysis, fresh soil was weighed before and after being dried for 1 h. To measure the electrical conductivity of soil water (ECsw1:5), soils were extracted in deionized water for 1 h to achieve a soil: water ratio of 1:5. The ECsw1:5 of the extract was subsequently measured using a conductivity meter. The available nitrate‐nitrogen and ammonia‐nitrogen, available phosphorus (AP), and available potassium (AK) in rhizosphere soil were determined according to the previous literature [52, 53]. Soil micronutrient concentrations were measured using the diethylenetriaminepentaacetic acid (DTPA) extraction method [54]. A total of 10 g of air‐dried soil was mixed with 20 mL of DTPA extraction solution (0.005 m DTPA, 0.01 m CaCl_2_, 0.1 m triethanolamine, pH 7.3) by shaking for 2 h at room temperature. The liquid supernatants were filtered and subsequently analyzed by inductively coupled plasma‐optical emission spectrometry (ICP‐OES, Optima 7300 DV, PerkinElmer) for calcium (Ca), magnesium (Mg) and sodium (Na) contents.

### 4.4 Quantitative PCR Analysis

Metagenomic sequencing and bioinformatics analysis identified key functional genes, which were further validated by qPCR and used to train machine learning models for disease prediction (Figure 1B,C). The primers were chosen for the determination of KO genes (Table S15). Soil DNA was extracted from 0.25 g of rhizosphere soil (collected from *A. sinensis*/*A. membranaceus* pots) using the DNeasy PowerSoil Pro Kit (Qiagen). Each PCR reaction, conducted in 8‐well tubes, utilized 20 µL of SYBR Premix Ex TaqTM (TaKaRa, Japan) for amplification of standard and DNA samples in qPCR. Each qPCR reaction included 2 µL of template DNA, 10 µL of SYBR Green Premix Ex Taq (2 ×), 0.4 µL of each primer, 0.4 µL of ROX Reference Dye II, and sterile water. qPCR followed the manufacturer's protocol: initial denaturation at 95°C for 3 min, followed by 40 cycles of denaturation at 95°C for 20 s, annealing at 62°C for 20 s, and extension at 72°C for 20 s, with a final extension step at 72°C for 10 min. Each sample was subjected to three replicate thermal cycling conditions, and the results were expressed as log copies/g^−1^ of dry soil according to standard procedures. The primer sequences, along with their corresponding melting curves and standard curve validation data, have been provided in Table S15 and Figures S8–S10. The abundance of *F. oxysporum* was quantified using a quantitative PCR (qPCR) assay targeting the *Fae* gene, which encodes ferulic acid esterase. This gene was selected because it is single‐copy in the *F. oxysporum* genome and exhibits high specificity for this species, enabling accurate quantification without cross‐reaction with other soil microbes [55, 56]. The primer sequences used were as follows: forward, 5'‐GGCATTTACTCCGCCACTTG‐3' and reverse, 5'‐AGCTCAGCGGCTTCCTATTG‐3' (Table S15).

### 4.5 Pot Experiment for Model Validation

To validate the predictive model under controlled conditions, we conducted a pot experiment was conducted using early‐stage soil samples collected from various fields in Gansu Province, a representative cultivation area for *Angelica sinensis* and *Astragalus membranaceus*. First, soil samples (n = 64) were collected from various fields and their microbial functional gene profiles were immediately analyzed via qPCR. This soil analysis constituted the “pre‐symptomatic” time point (T0). Subsequently, the same characterized soils were used in a pot experiment to grow *Angelica sinensis* and *Astragalus membranaceus*. Seeds of *A. sinensis* (n = 32 replicates) and *A. membranaceus* (n = 32 replicates) were sown in these soils. The experiment was terminated and the final plant health status (healthy or root rot symptomatic root) was meticulously recorded at the stage when widespread and severe symptomatic disease was manifest in the population. Finally, the final disease outcome of each plant was recorded and used as the ground truth for model validation.

### 4.6 Machine Learning

To develop a predictive model for root rot disease based on microbial functional gene abundance, we employed seven machine learning algorithms using the scikit‐learn (sklearn) library (version 1.2.2) in Python [57]. The algorithms included Support Vector Machine (SVM), Decision Tree Classifier, K‐Nearest Neighbors (K‐NN), AdaBoost Classifier, Logistic Regression, Random Forest, and Multilayer Perceptron Classifier (MLP). The dataset consisted of qPCR abundance measurements of three key biomarker genes (K18968, K15011, K13290) obtained from 199 rhizosphere soil samples. These samples were randomly partitioned into a training set (70%) and an independent testing set (30%) using a stratified sampling approach to maintain class distribution. We performed 5‐fold cross‐validation on the training set for hyperparameter tuning and model selection. Model performance was evaluated using the area under the receiver operating characteristic curve (AUC), accuracy, sensitivity, and specificity. The optimal hyperparameters for each model were determined through grid search with 5‐fold cross‐validation. The final model was selected based on the highest AUC value from the test set. A comprehensive summary of performance metrics (including Accuracy, F1, Recall, Precision, and ROC AUC) for all seven models on both training and testing datasets is provided in Table S13. To benchmark the predictive performance of genetic markers against traditional soil assays, we constructed a comparative model using nine measured soil physicochemical parameters: pH, cation exchange capacity (CEC), soil moisture, nitrate‑N (NO_3_^−^─N), ammonium‑N (NH_4_^+^─N), available phosphorus (AP), available potassium (AK), calcium (Ca), magnesium (Mg), and sodium (Na). The same machine‑learning workflow employed for the genetic marker model was applied, including identical train/test split (70%/30%, stratified), the same seven algorithms (SVM, Decision Tree, K‑NN, AdaBoost, Logistic Regression, Random Forest, and MLP), 5‑fold cross‑validation for hyperparameter tuning, and consistent evaluation metrics (AUC, accuracy, sensitivity, specificity). This design enabled a direct comparison of model performance between genetic and physicochemical feature sets. Results of this benchmarking analysis are presented in Figure S8 and Table S14.

### 4.7 Statistical Analysis

All statistical analysis was conducted using R (version 4.2.1). Detailed descriptions of the statistical methods are provided in figure legends. Data visualization was performed using the “ggplot2” package (version 3.3.5). Group differences in microbial gene abundance between healthy and diseased samples were assessed using the Wilcoxon rank‐sum test for non‐normally distributed data. Weighted Gene Co‐expression Network Analysis (WGCNA) was performed using the WGCNA R package (version 1.72–1) to identify co‐expression modules of KEGG Orthology (KO) genes. Significant Temporal Expression Analysis (STEMP) was implemented using custom R scripts (version 4.2.1) incorporating functions from the stats (version 4.2.1) and multcomp (version 1.4–25) packages. Correlation analysis between metagenomic and qPCR‐derived gene abundances was performed using Spearman's rank correlation.

## Conflicts of Interest

The authors declare no conflict of interest.

## Supporting information




**Supporting File**: advs74412‐sup‐0001‐SuppMat1.docx.

**Supporting File**: advs74412‐sup‐0001‐SuppMat2.xlsx.

## Data Availability

Metagenomic raw data used in this study are deposited in the National Genomics Data Center (NGDC) database under BioProject PRJCA043663, with accession number: subCRA049153. Other raw data is available on request.
