# Supplementary material for: Forecasting Root Rot Disease through Predictive Microbial Functional Profiling
Source: Adv Sci (Weinh). 2026 Feb 17;13(23):e22628. doi: 10.1002/advs.202522628 (PMC13104130; doi:10.1002/advs.202522628)
Supplement: Supplementary file 1 — Supporting File: advs74412‐sup‐0001‐SuppMat1.docx. [file ADVS-13-e22628-s002.docx]

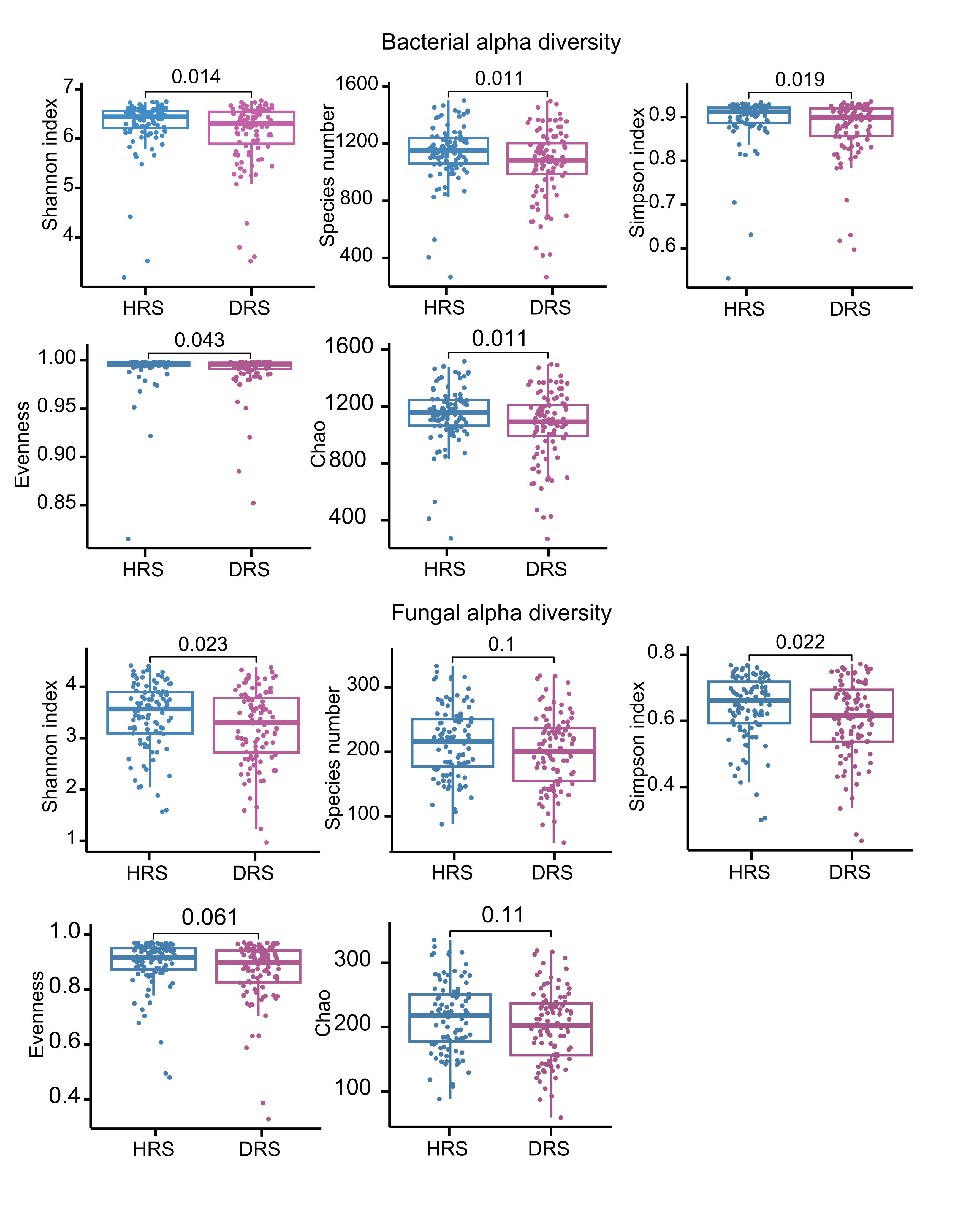
**Figure S1.** Box plot of rhizosphere bacterial and fungal alpha diversity (Shannon, Species number, Simpson, Evenness and Chao1) in healthy (n = 99) and diseased (n = 100) samples; p-values from wilcoxon test were shown above each panel.


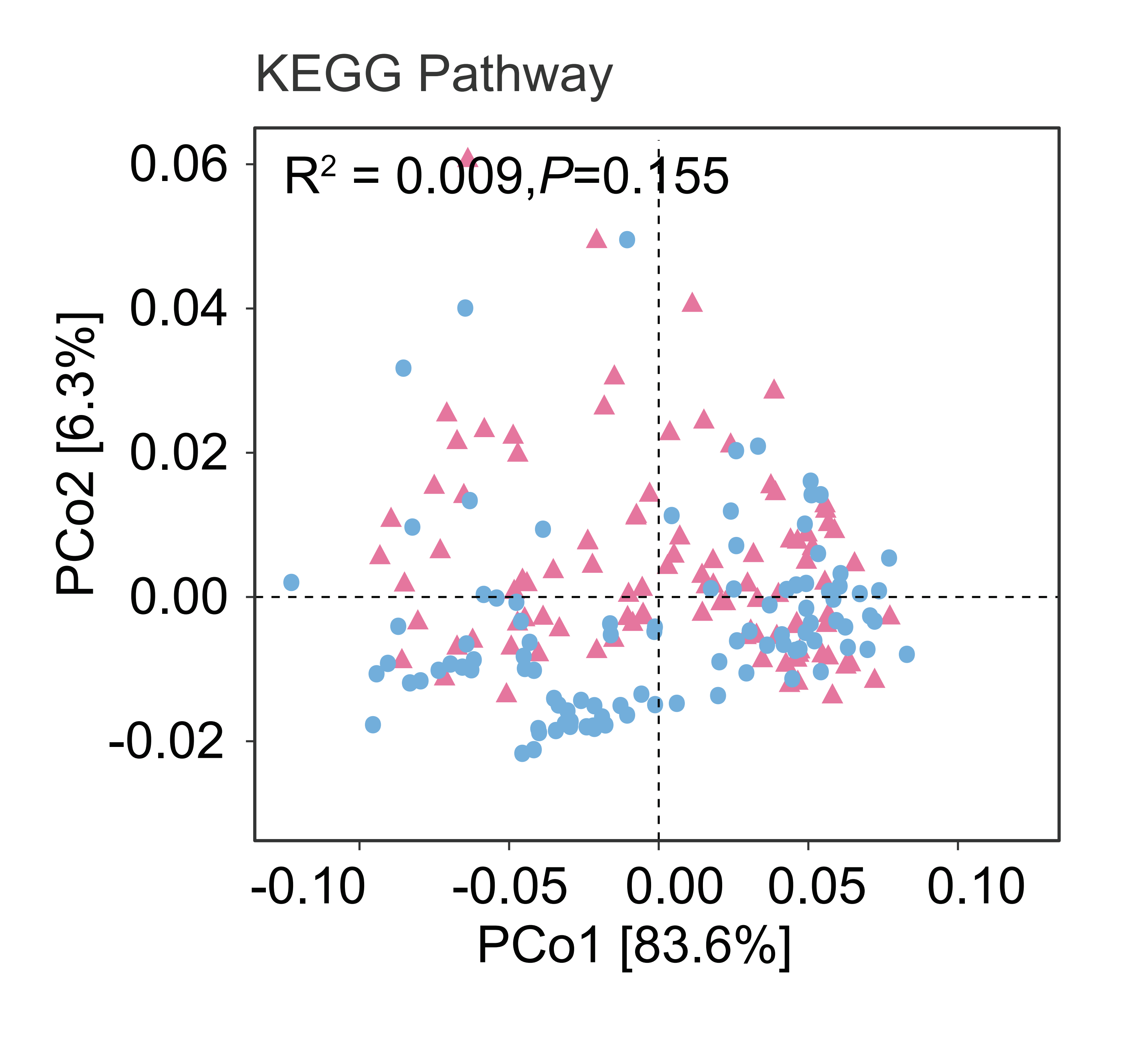


**Figure S2.** Principal coordinates analysis (PCoA) based on Bray–Curtis distances showing significant separation between healthy (n = 99) and diseased (n = 100) samples of KEGG pathways. Percentages of variance explained (R²) and p-values from ANOVA are indicated for each component.


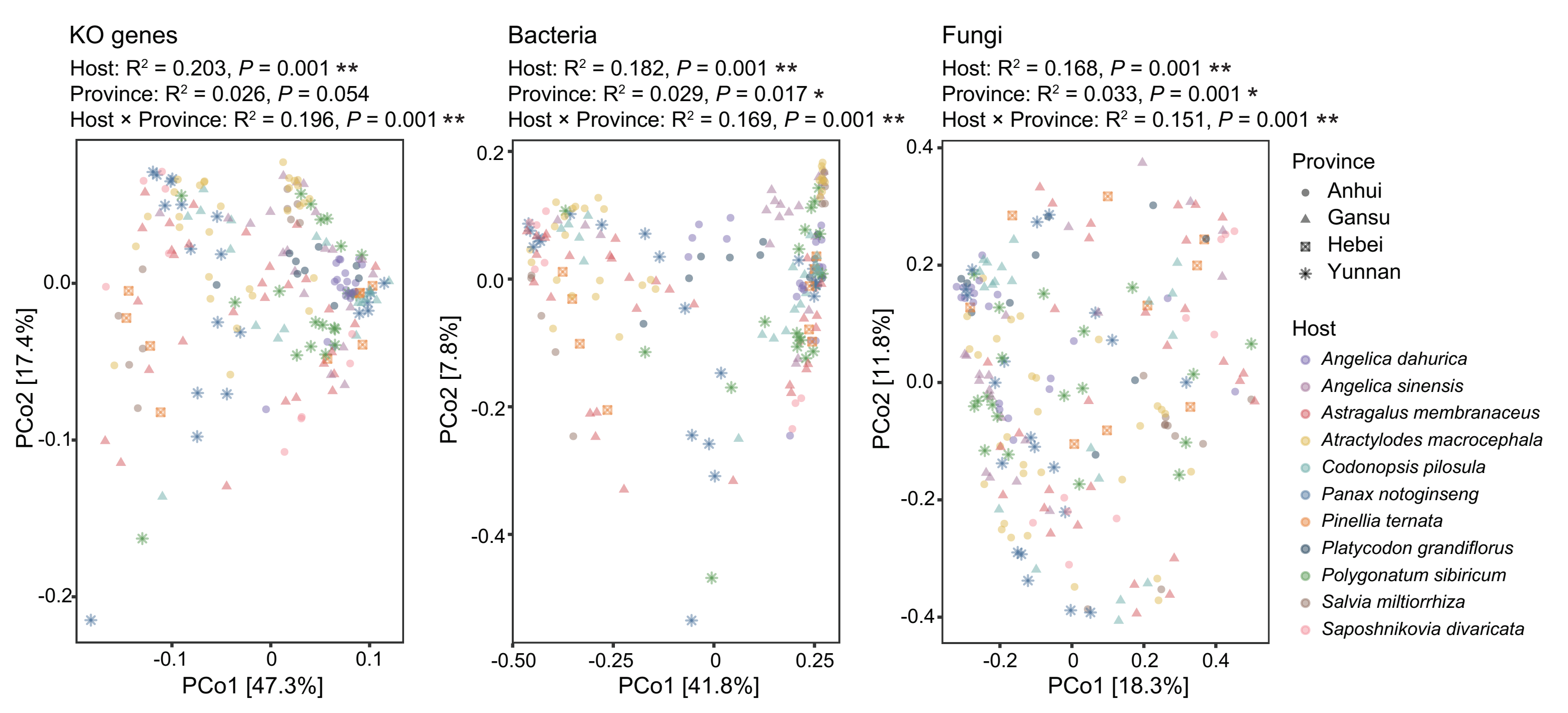


**Figure S3.** Principal coordinates analysis (PCoA) based on Bray–Curtis distances showing significant separation among host and province samples across bacterial community, fungal community, and KO gene profiles.


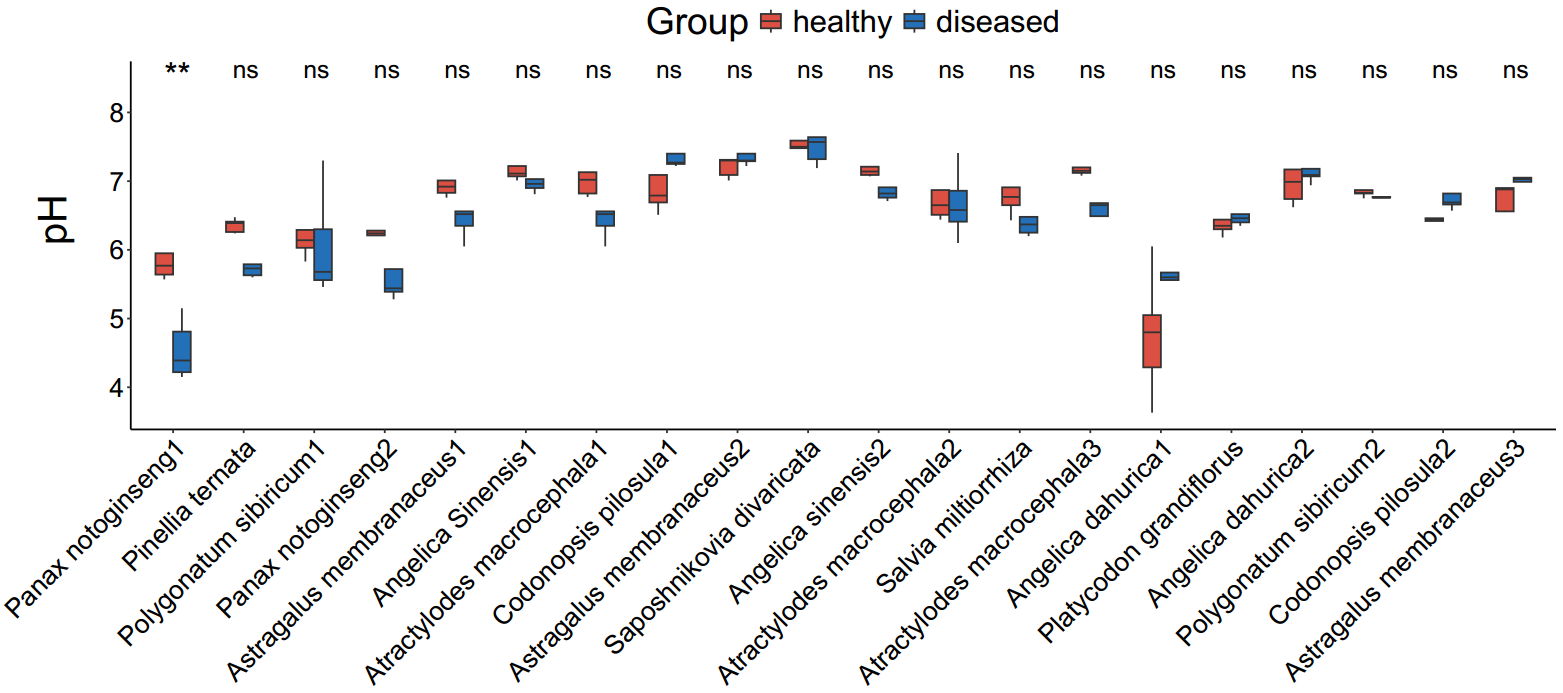

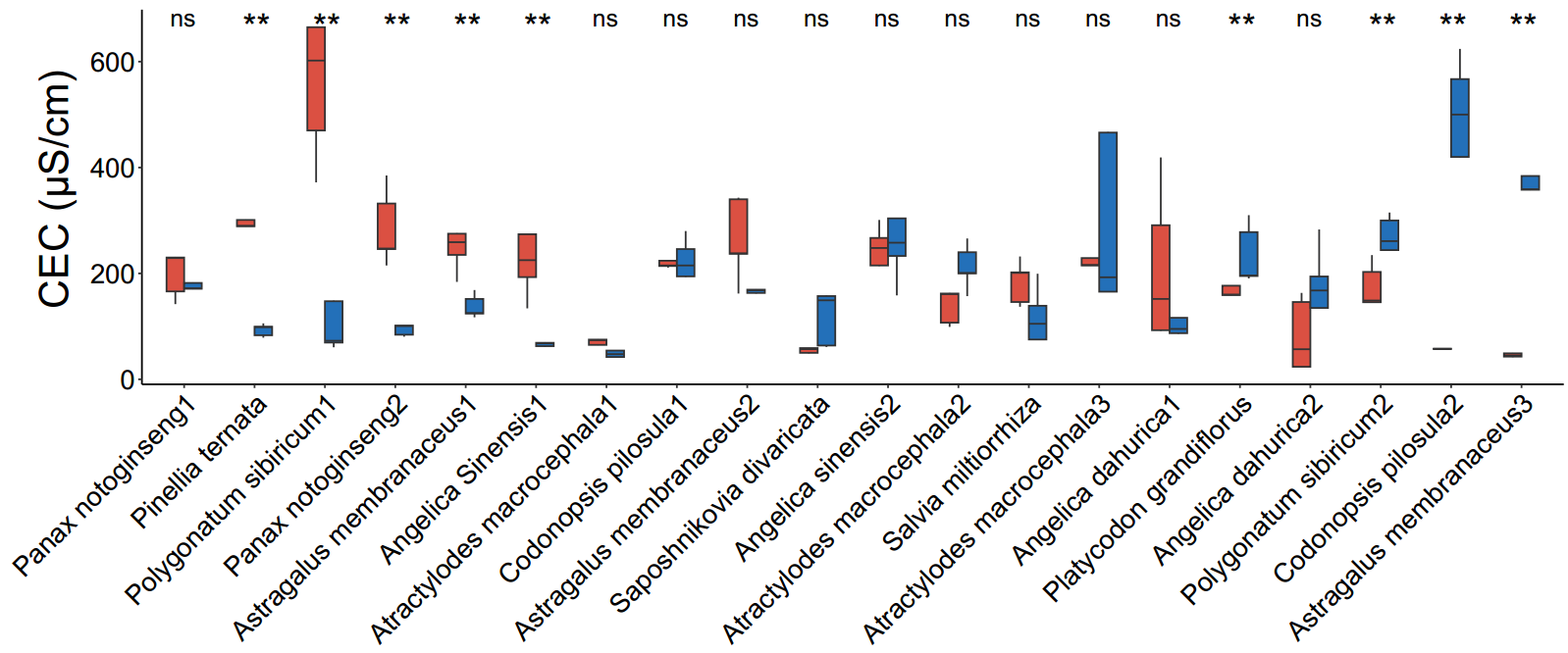

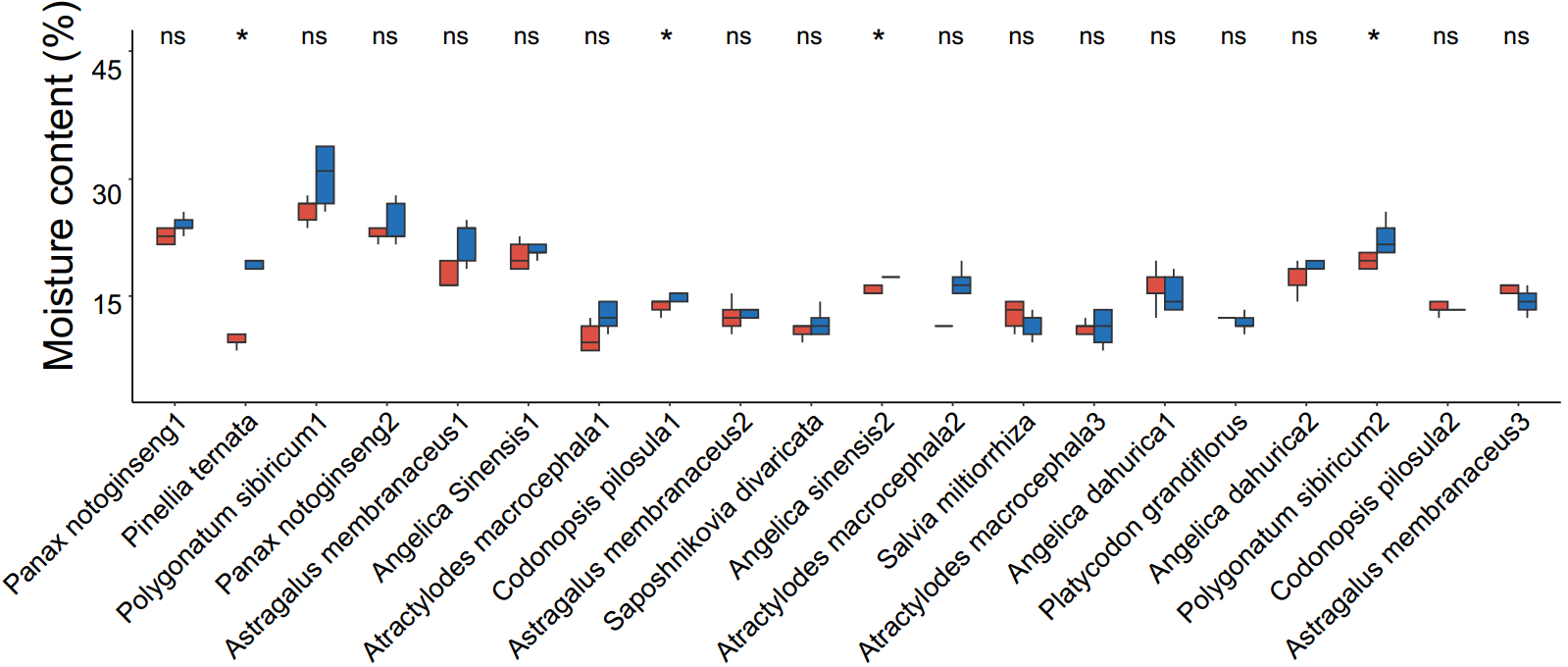

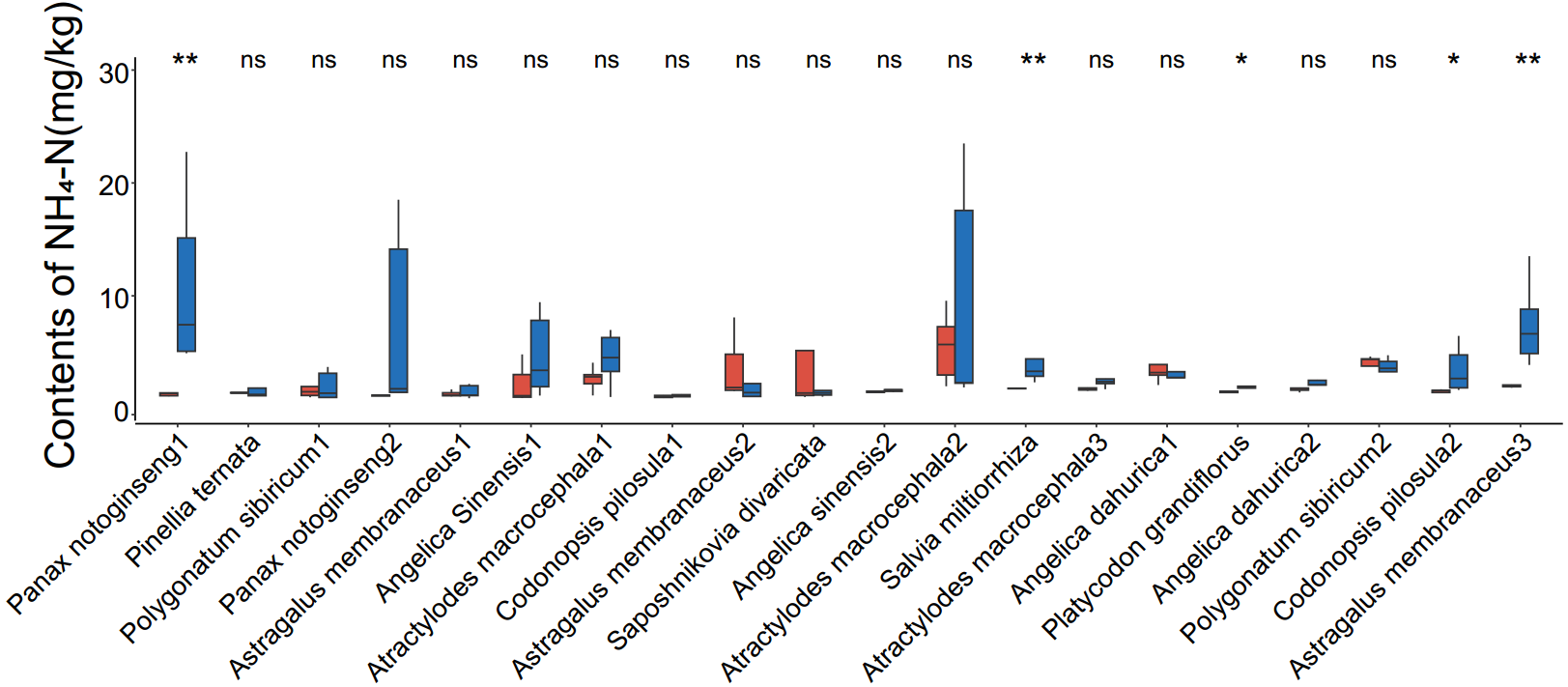


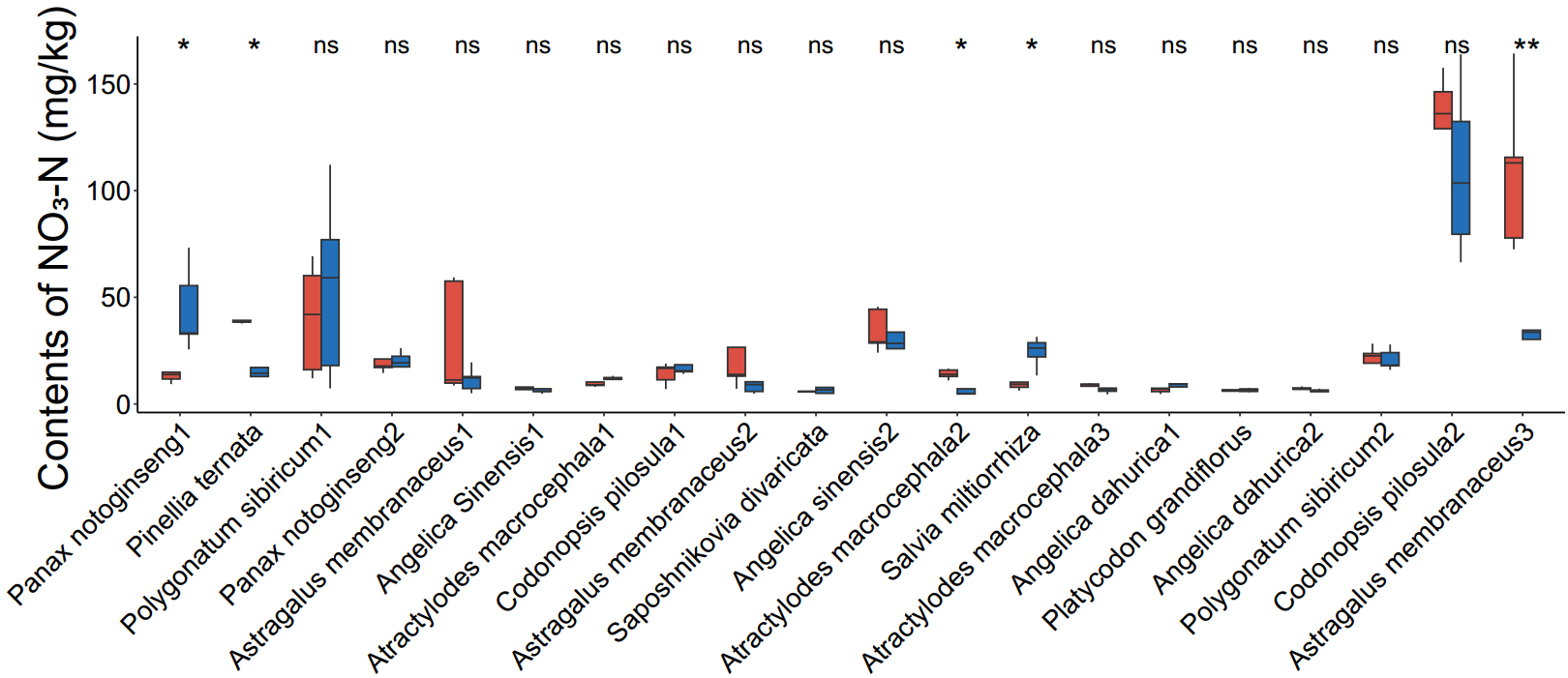

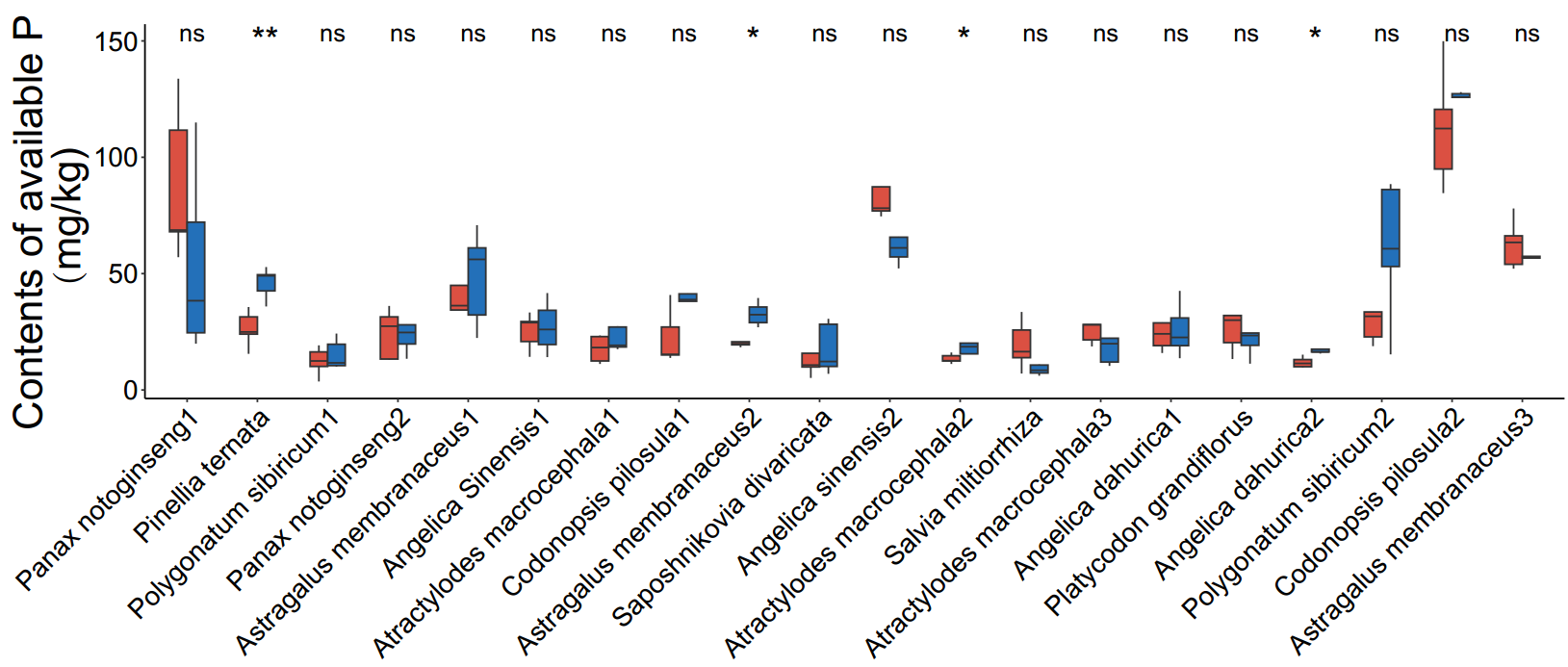

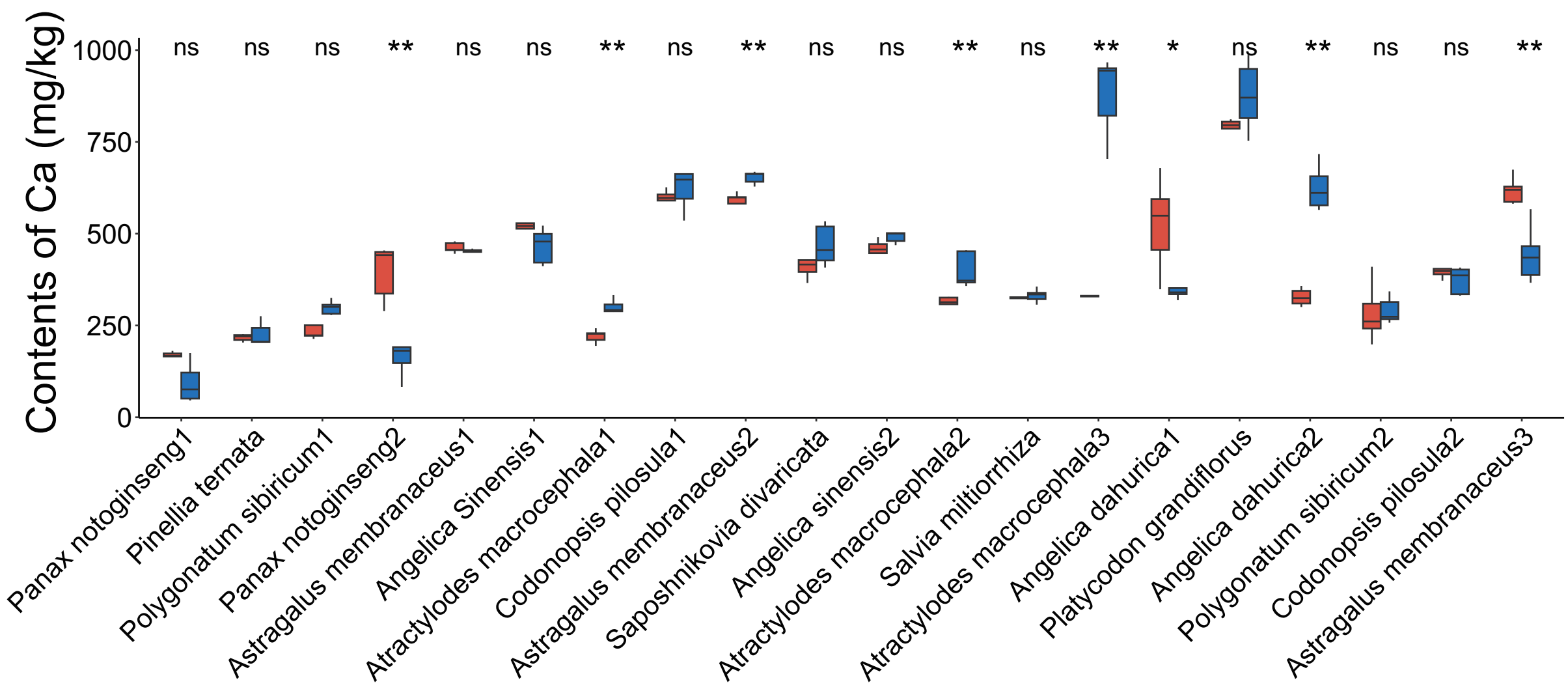

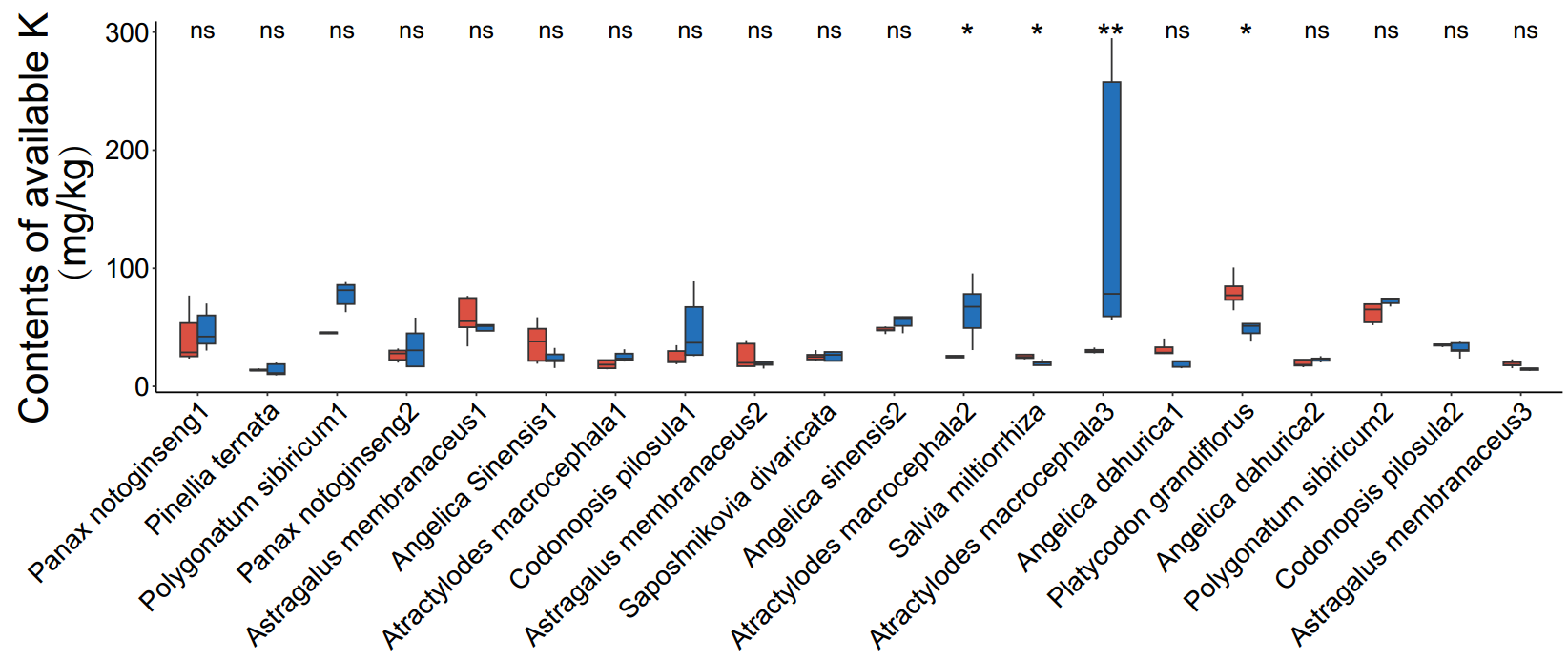

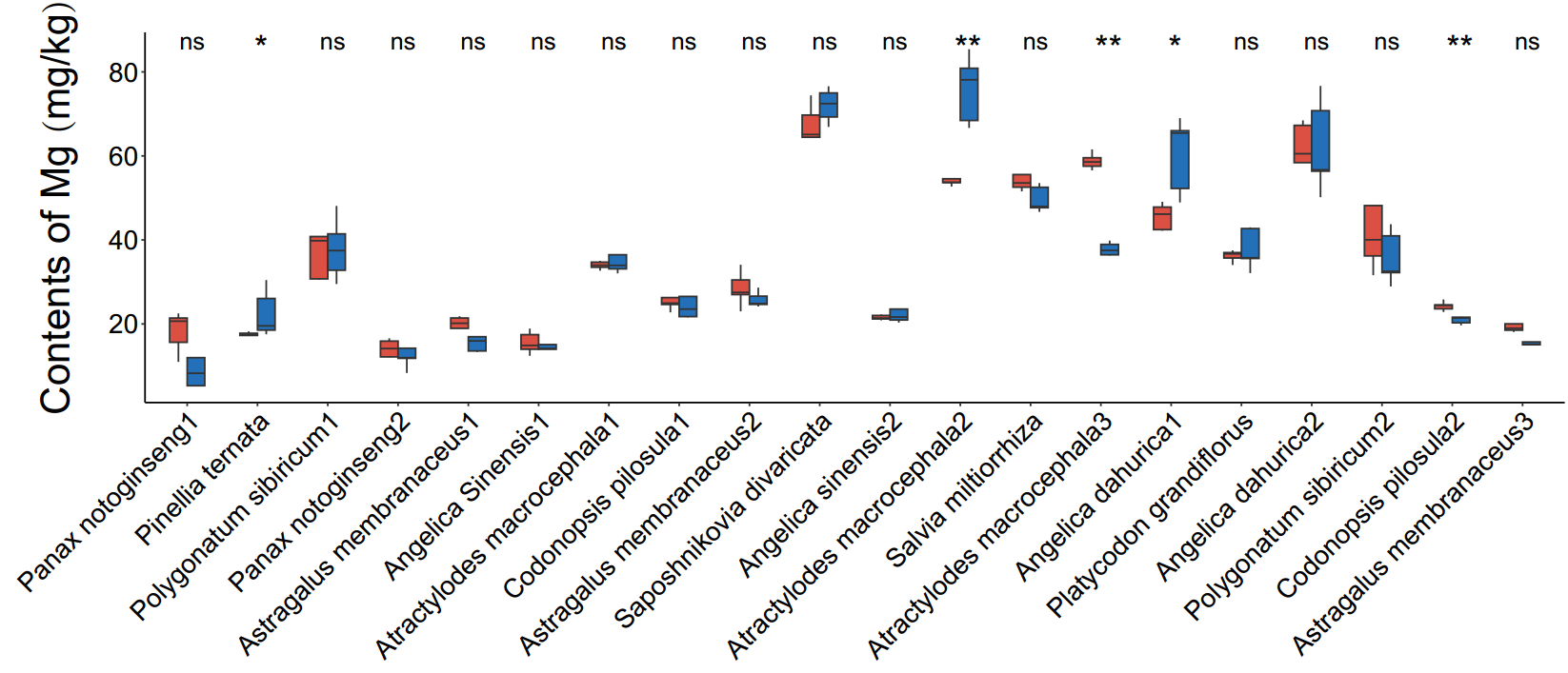

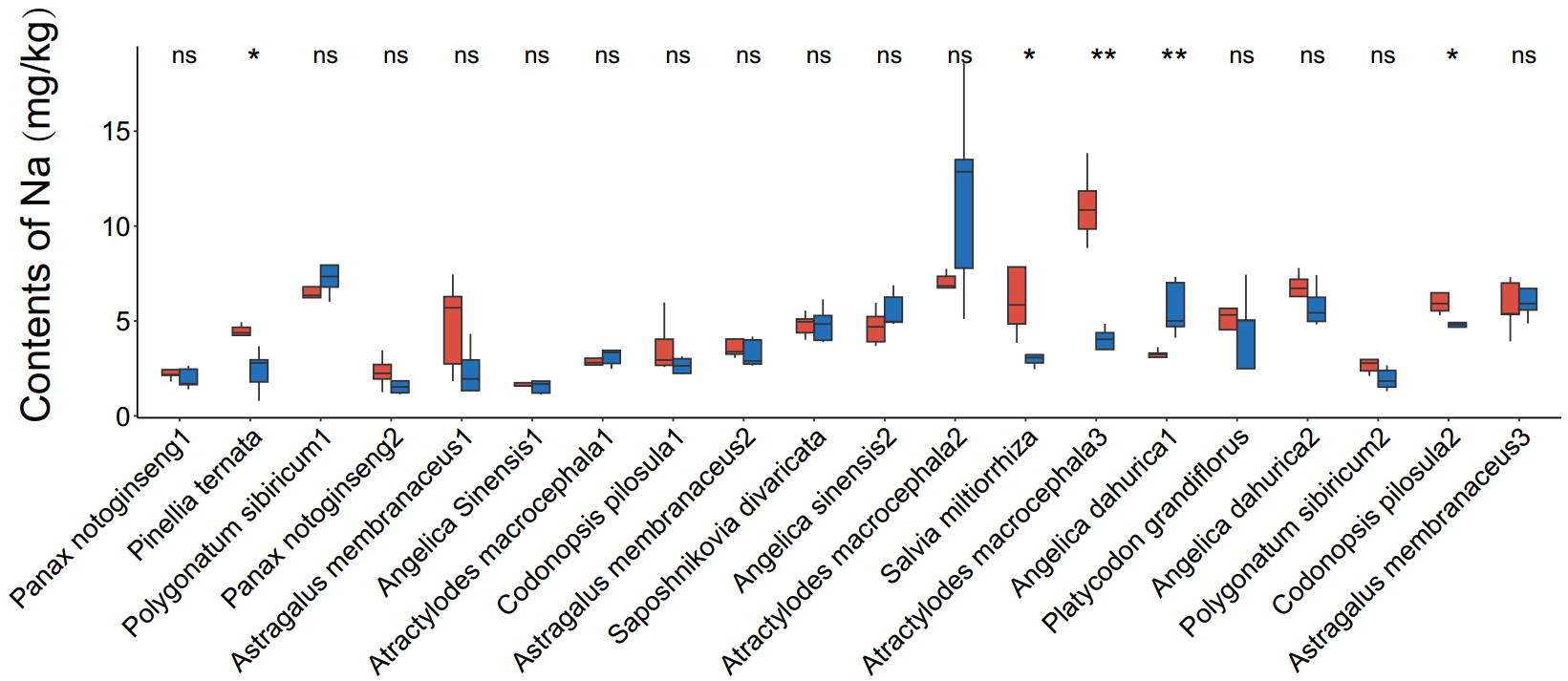


**Figure S4.** Physicochemical properties of healthy and diseased rhizosphere soils. Boxplots comparing the distribution of various soil physicochemical properties between healthy (n = 99) and diseased (n = 100) rhizosphere samples. Statistical significance was determined by the Wilcoxon rank-sum test and is denoted by asterisks: *P < 0.05, **P < 0.01, ***P < 0.001.


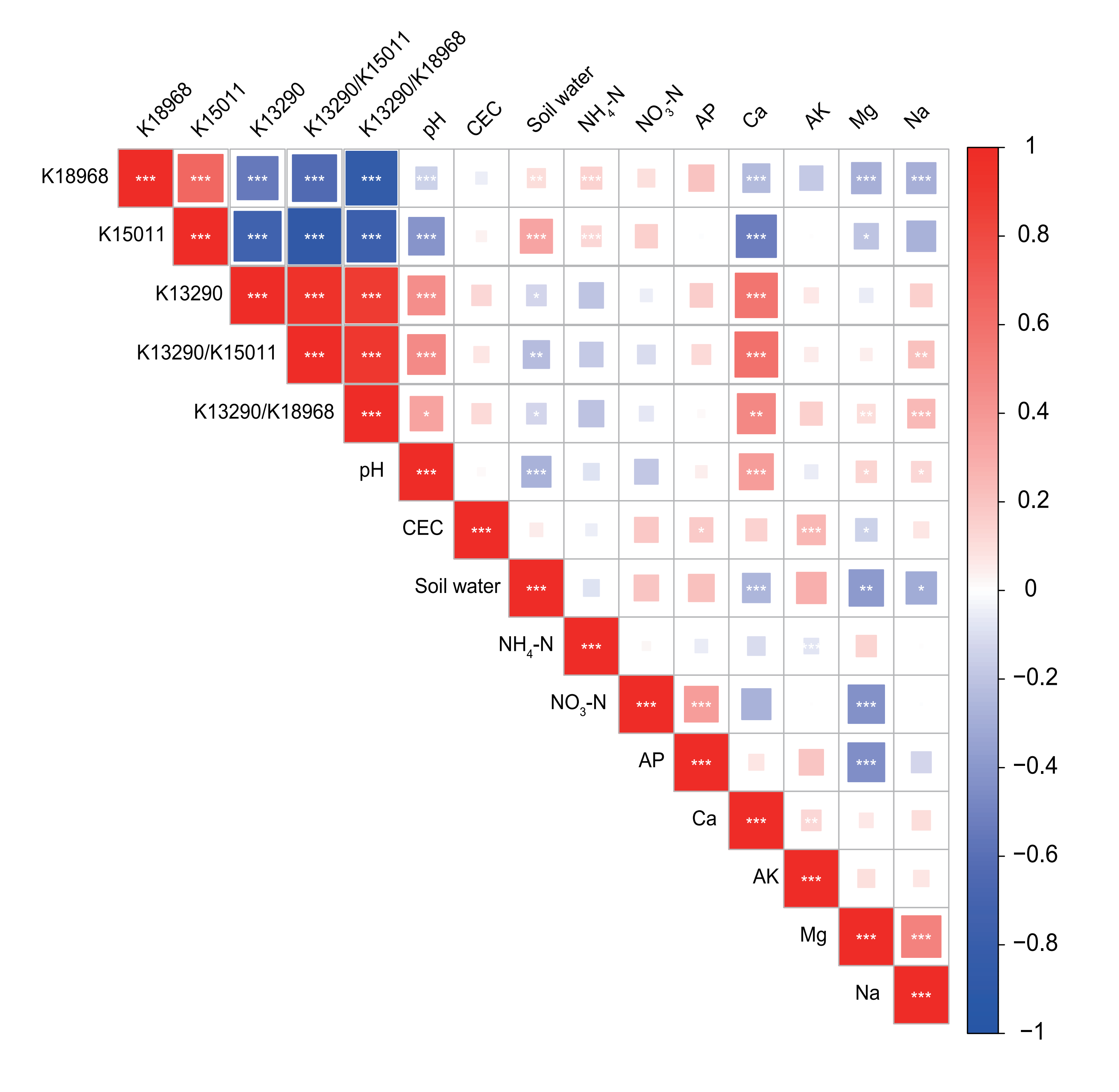


**Figure S5.** Correlation between three key rhizosphere microbial functional genes and the rhizosphere soil physicochemical properties. The color depth and size of the dots show the strength of the correlation (R value). The darker the red is, the stronger the positive correlation is. The darker the blue is, the stronger the negative correlation is. The white represents no correlation. The larger the dot is, the stronger the correlation is. *P < 0.05; **P < 0.01; ***P < 0.001. The ratios K13290/K15011 and K13290/K18968 were calculated to illustrate the relative changes in abundance between the health-associated gene (K13290) and the two disease-associated genes (K15011 and K18968). These ratios serve to visualize the functional imbalance between beneficial and pathogenic metabolic potentials in the rhizosphere.


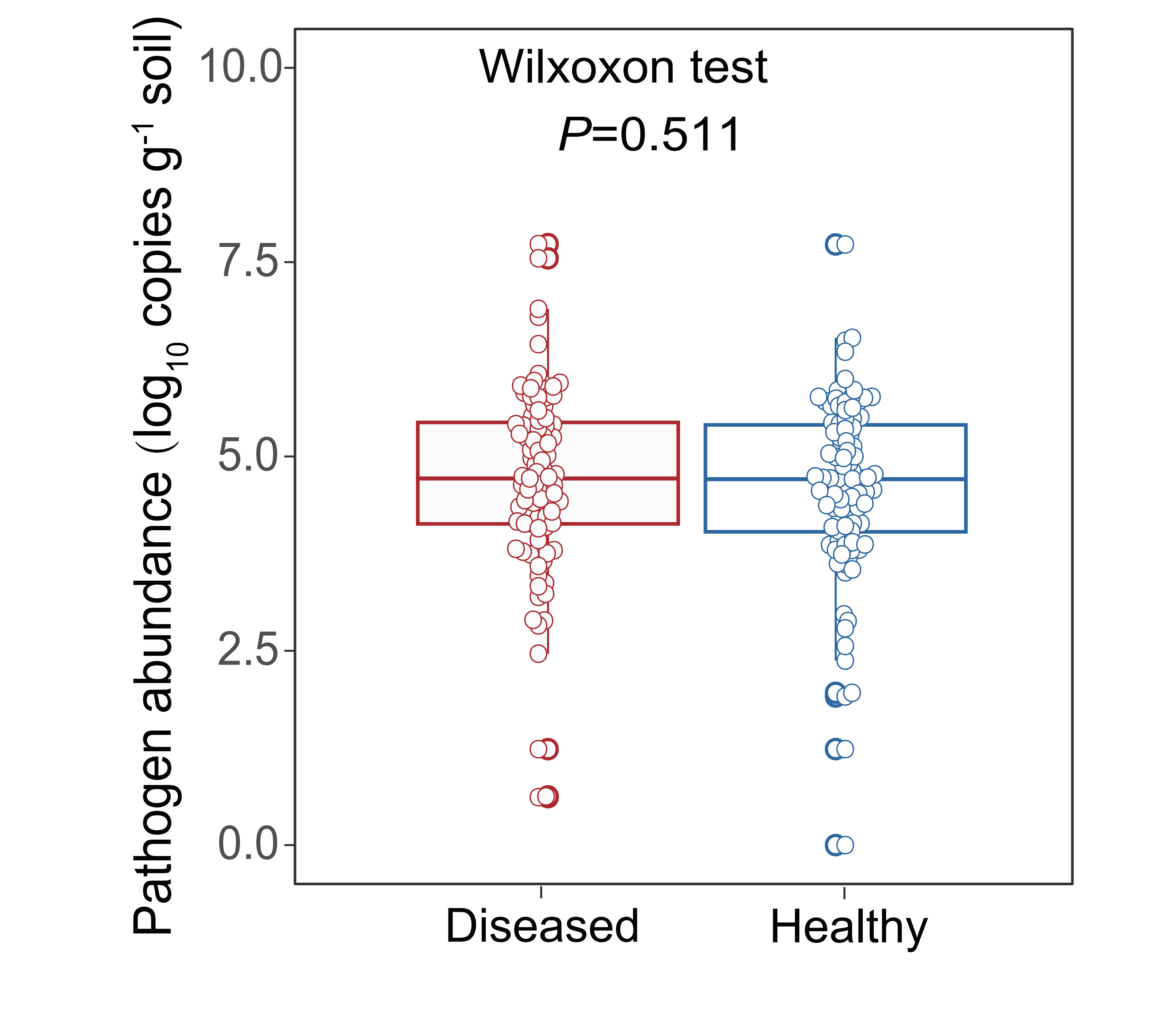


**Figure S6.** *F. oxysporum* gene copy numbers in diseased (n = 100) and healthy (n = 99) rhizosphere soils.


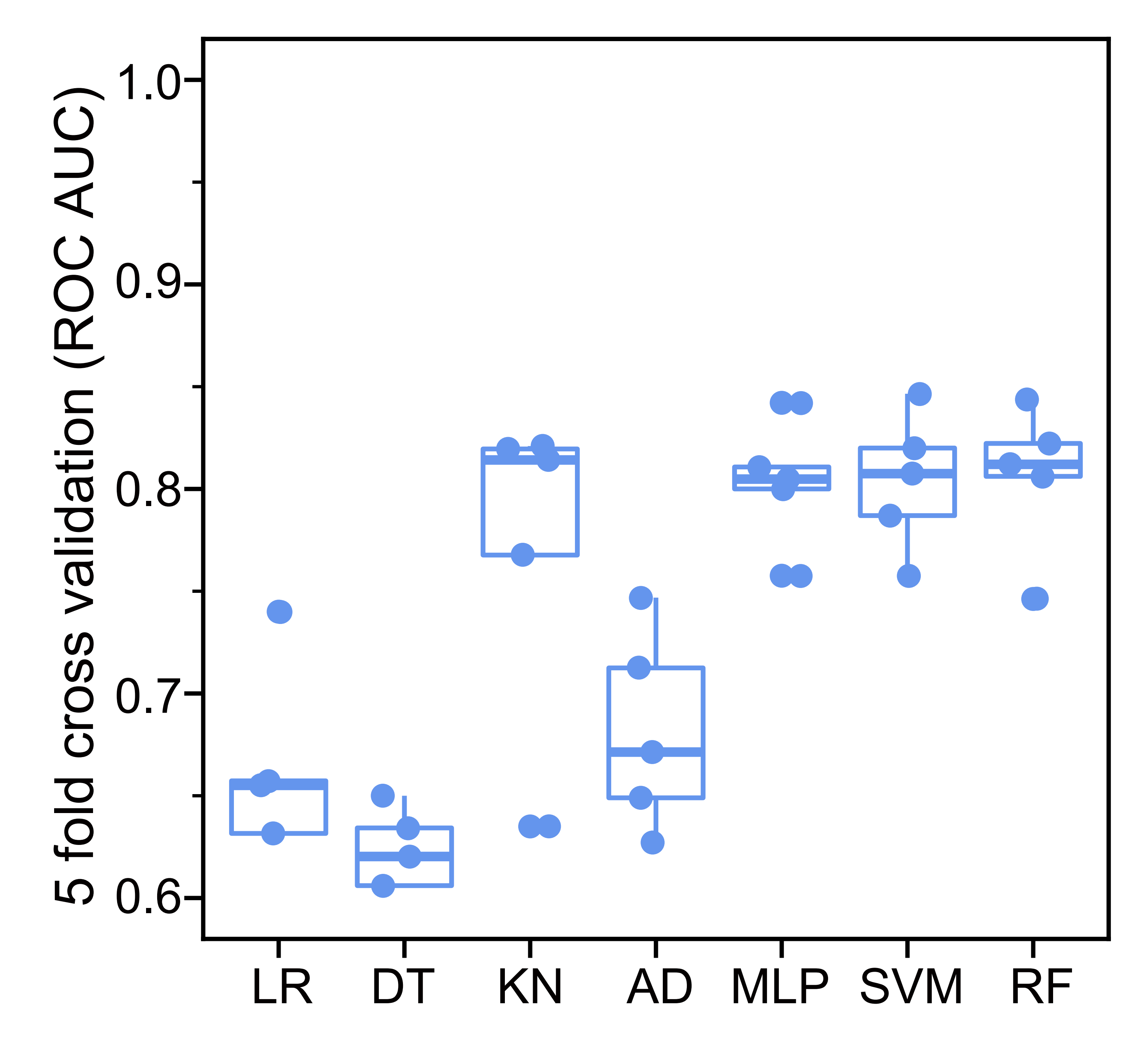


**Figure S7.** Model comparison on the hold-out test set. Receiver Operating Characteristic (ROC) curves for the seven machine learning models trained on soil physicochemical properties data (n=199 samples, 7:3 split). LR: Logistic Regression, DT: Decision Tree, KN: K-Nearest Neighbors, AD: AdaBoost, MLP: Multilayer Perceptron, SVM: Support Vector Machine, RF: Random Forest.

**
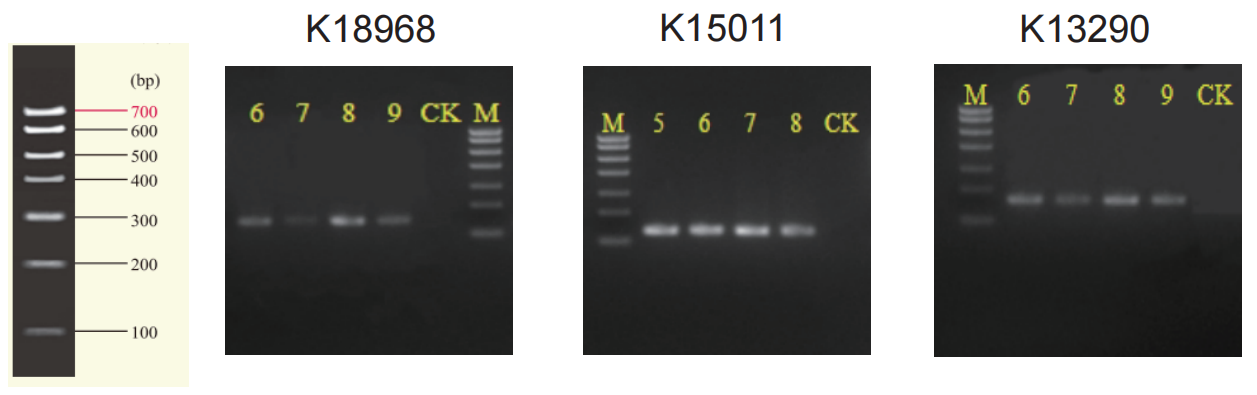
**

**Figure S8.** Agarose gel electrophoresis of purified PCR products on 2% agarose gel from partial samples.


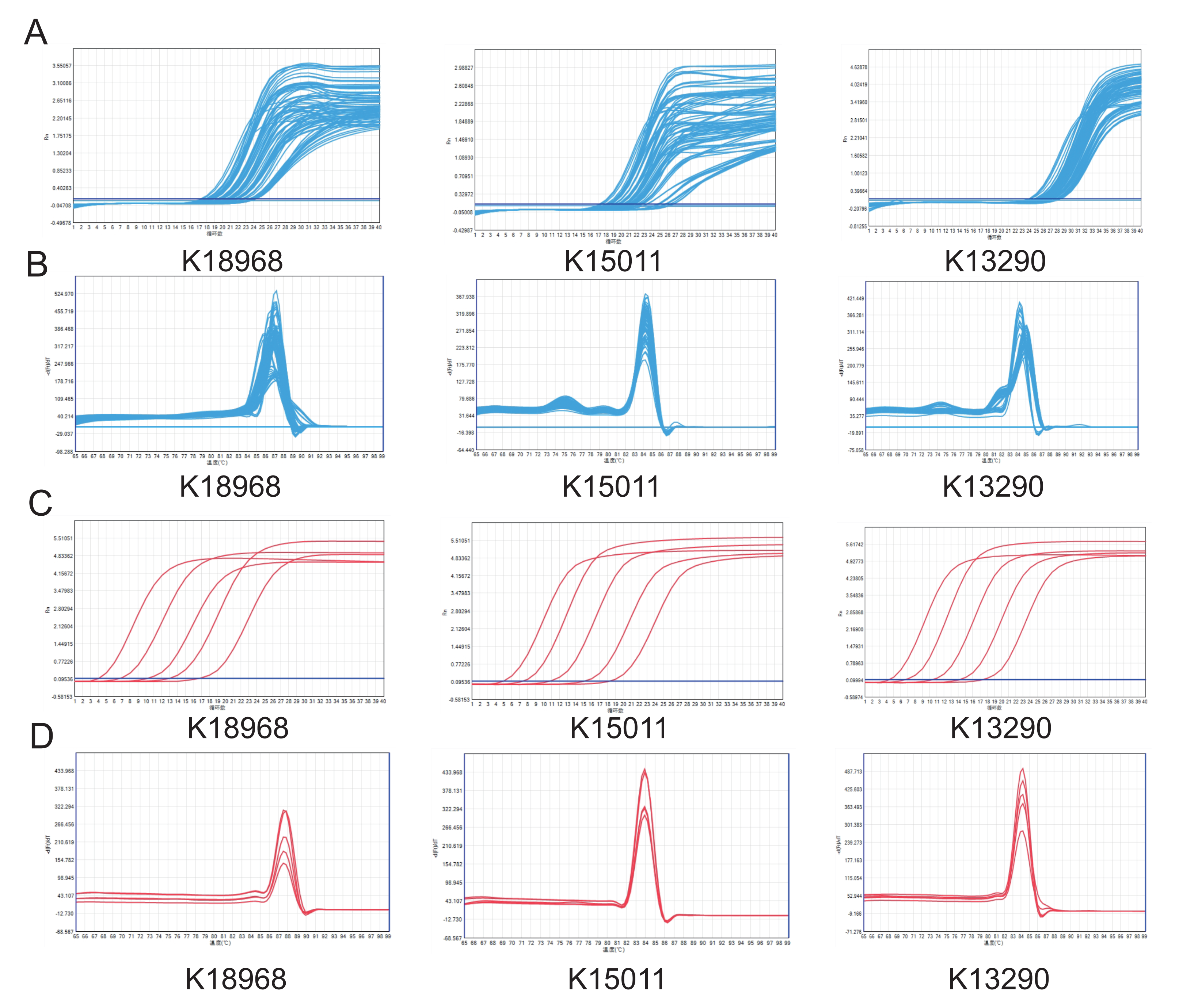


**Figure S9. A.** The fluorescence quantitative PCR amplification curve of the samples. **B.** The fluorescence quantitative PCR melting curve of the samples. **C.** The fluorescence quantitative PCR amplification curve of the gene standard. **D.** The fluorescence quantitative PCR melting curve of the gene standard.

**
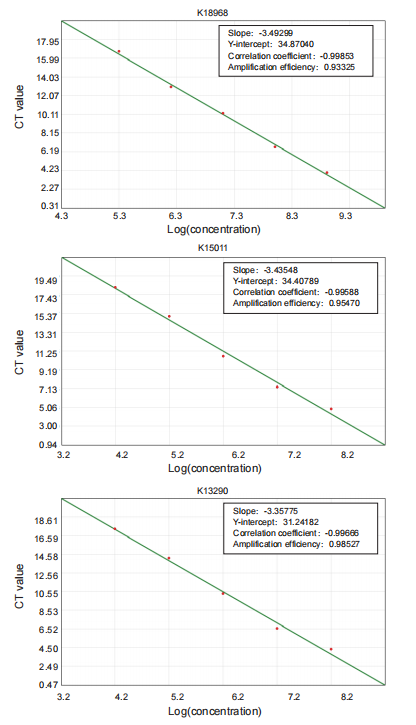
**

**Figure S10.** The standard curve of the genes. CT: Cycle Threshold.
